# Drivers of Variation in Health Care Spending Across US Counties

**DOI:** 10.1001/jamahealthforum.2024.5220

**Published:** 2025-02-14

**Authors:** Joseph L. Dieleman, Maxwell Weil, Meera Beauchamp, Catherine Bisignano, Sawyer W. Crosby, Drew DeJarnatt, Haley Lescinsky, Ali H. Mokdad, Samuel Ostroff, Hilary Paul, Ian Pollock, Maitreyi Sahu, John W. Scott, Kayla V. Taylor, Azalea Thomson, Marcia R. Weaver, Lauren B. Wilner, Christopher J. L. Murray

**Affiliations:** 1 Institute for Health Metrics and Evaluation, Hans Rosling Center for Population Health, Seattle, Washington; 2 Department of Surgery, Division of Trauma, Burn & Critical Care Surgery, Harborview Medical Center, Seattle, Washington

## Abstract

**Importance:**

Understanding the drivers of health care spending across US counties is important for developing policies and assessing the allocation of health care services.

**Objective:**

To estimate the amount of cross-county health care spending variation explained by (1) population age, (2) health condition prevalence, (3) service utilization, and (4) service price and intensity.

**Design, Setting, and Participants:**

In this cross-sectional study, data for 4 key drivers of per capita spending were extracted for 3110 US counties, 148 health conditions, 38 age-sex groups, 4 payers, and 7 types of care for 2019. Service utilization was measured as service volume per prevalent case, while price and intensity was measured as spending per visit, admission, or prescription. Das Gupta and Shapley decomposition methods and linear regression were used to estimate the contribution of each factor. The data analysis was conducted between March 2024 and July 2024.

**Exposures:**

Age, disease prevalence, service utilization, or service price and intensity.

**Main Outcomes and Measures:**

Variation in health care spending across US counties.

**Results:**

In 2019, 76.6% of personal health care spending was included in this study. Overall, 64.8% of cross-county health care spending variation among 3110 US counties was explained by service utilization, while population age, disease prevalence, and price and intensity of services explained 4.1%, 7.0%, and 24.1%, respectively. The rate at which these factors contributed to variation in spending differed by payer, type of care, and health condition. Service utilization was associated with insurance coverage, median income, and education. An increase in each of these from the median to the 75th percentile was associated with a 7.8%, 4.4%, and 3.8% increase in ambulatory care utilization, respectively. The fraction of Medicare beneficiaries with Medicare Advantage was associated with less utilization. An increase in Medicare Advantage coverage from the median to the 75th percentile was associated with a 1.9% decrease in ambulatory care utilization. Differences in cross-state spending levels were also attributed to different factors. For Utah, the state with the least health care spending per capita, spending rates were lower for all types of care due principally to the young age profile. For New York, the state with the highest spending, spending rates were relatively high for hospital inpatient and prescribed pharmaceutical spending. For both types of care, high service price and intensity contributed to the above-average spending.

**Conclusions and Relevance:**

In this cross-sectional study, variation in health care spending among US counties was largely related to variation in service utilization. Understanding the drivers of spending variation in the US may help policymakers assess the allocation of health care resources.

## Key Points

**Question:**

What factors explain variation in health care spending across US counties?

**Findings:**

In this cross-sectional study of health care spending from 3110 US counties in 2019, 65% of the cross-county variation was explained by variation in service utilization, while price and intensity of services, disease prevalence, and population age explained 24%, 7%, and 4% of the variation, respectively. Insurance coverage and median income were associated with service utilization, while the fraction of Medicare with Medicare Advantage was associated with less utilization.

**Meaning:**

Understanding the key factors that explain the variation in health care spending across the US is valuable for developing health care policies and assessing the allocation of health care resources.

## Teaser

This cross-sectional study estimates the amount of cross-county health care spending variation explained by population age, health conditions prevalence, service utilization, and service price and intensity.

## Introduction

While the US as a whole spends a substantial amount on health, there is wide variation in spending levels across US states.^1,2^ In Dieleman et al,^3,4^ we showed that some US counties spent more than 5 times as much per person as other counties, even after adjusting for age. Characterizing drivers of this regional variation in commercial, public, and out-of-pocket spending can help to inform health policy.

A few studies have examined drivers of variation in spending across US regions. For example, a National Academies of Medicine consensus report highlighted that Medicare’s spending varied mostly because of differences in the number and which types of services are provided to patients.^5^ Cutler et al^6^ found that regional differences in Medicare expenditure are determined more by clinician beliefs than patient demand. Other researchers (Fisher et al^7^ and Finkelstein et al^8^) found that geographic variations in Medicare spending were caused by supply-and-demand dynamics between patients and physicians.^7,8^ Cooper et al^9,10^ looked at geographic variation in regional health spending in the US for Medicare, Medicaid, and private insurance and found that spending per beneficiary for different payers was not correlated geographically.

However, several key gaps still remain in the existing scientific literature. First, the majority of prior research focuses on Medicare, but Medicare was only 26% of personal health care spending in 2022, and spending by other funders focuses on different health conditions and types of care.^4,11^ Second, although disease patterns, population characteristics, access to health care, and use of services are all known to vary locally, existing research on geographic variation in health care spending has focused mainly on state variation rather than within-state county-level variation. Third, existing research has generally not used disease-specific spending estimates and therefore could not control for disease prevalence. To our knowledge, no study has assessed within-state total and payer-specific spending (for all major payers), controlling for disease prevalence.

We address these limitations simultaneously by assessing variation in health condition–specific and payer-specific spending across US counties. Specifically, we estimated the contribution of 4 key drivers of spending on rates of health care spending for 4 payer categories, 7 types of service, and 148 health conditions across 38 age-sex groups and 3110 US counties for 2019. In addition, we assessed the factors associated with differences in utilization rates and the price and intensity of care.

## Methods

This study was reviewed and approved by the University of Washington institutional review board; because data were deidentified, informed consent was waived. The study complies with the Guidelines for Accurate and Transparent Health Estimates Reporting (GATHER) statement (eAppendix in Supplement 1).^12^

The conduct of this cross-sectional study followed 4 main steps. First, we extracted 2019 estimates from 3 major databases. From the US Disease Expenditure project, we extracted personal health care spending and service utilization estimates for each US county and state, age and sex group, health condition, type of care, and payer.^4^ Personal health spending is all health spending on an individual and excludes spending on public health and research and development. In this study, service utilization is the number of visits, admissions, or prescriptions per incident or prevalent case. From the US Health Disparities (USHD) project, we extracted estimates of mortality by cause of death at the county level by age and sex.^13^ Additionally, from the Global Burden of Disease (GBD) 2021 study, we extracted 2019 estimates of mortality, incidence, and prevalence by cause at the state level by age and sex.^14,15^ Second, we estimated incidence (for all injuries and cancers) or prevalence (for all other conditions) using linear regression and data from the GBD 2021 study and USHD. (Hereafter, we refer to incidence and prevalence simply as prevalence, but for injuries and cancer, incidence was always used.) Third, we used decomposition methods to assess factors that explain the cross-county variation in spending levels. These methods explored the contribution of (1) the age and sex profile of the population, (2) health condition prevalence rates, (3) service utilization, and (4) service price and intensity toward explaining spending variation. Service price and intensity was defined as spending per visit, per admission, or per prescription. Fourth, we used linear regression methods to identify factors associated with service utilization and service price and intensity.

### Step 1: Data Extraction

#### US Health Care Spending and Utilization Data

The spending and utilization estimates extracted from the Disease Expenditure Project were stratified by 3110 US counties, 148 health conditions, 38 age and sex groups, 7 type-of-care categories, and 4 payer categories. The spending and utilization estimates we used for this study are available for download from the Global Health Data Exchange.

To generate those spending and utilization estimates, the Disease Expenditure 2019 project assessed more than 40 billion insurance claims, with more than 800 million from hospital records, survey data, and government estimates. These data included Medicare claims, Medicaid claims, commercial claims from the Health Care Cost Institute, Kythera, MarketScan, Fluent Dental Strategies, and hospital administrative data sources from the Agency for Healthcare Research and Quality Healthcare Cost and Utilization Project series. Household survey data from the Medical Expenditure Panel Survey were also extracted. These data were parsed into health system encounters, visits, admissions, and prescriptions, which often include more than 1 claim, and assigned a primary diagnosis based on spending amounts. All diagnoses were mapped to 1 of 148 health conditions based on methods initially derived for the GBD study.^16^ Statistical methods were used to adjust for data imperfections, such as the use of zip codes rather than counties or the use of nature of injury codes rather than codes that identify the cause of the injury; address small data concerns associated with having incomplete underlying data spread across many counties and health conditions; and estimate uncertainty. Finally, these estimates were adjusted for the presence of comorbidities using linear regressions, such that the resulting estimates track spending attributable to each health condition, and were scaled to align with the official government spending estimates.^2,17^ Estimates of pharmaceutical spending exclude the cost of pharmaceutical rebates and discounts.

#### USHD Mortality Data

We used estimates of cause-specific mortality by US county from the USHD study.^13^ These USHD mortality estimates were produced using deidentified death records from the US National Vital Statistics System and population estimates from the US National Center for Health Statistics. The death records data were tabulated by cause of death, county, age group, sex, and year. Causes of death were mapped from the *International Statistical Classification of Diseases, Tenth Revision, Clinical Modification (ICD-10-CM)* codes.^18^ The USHD county-specific mortality estimates we extracted for this study are also available for download from the Global Health Data Exchange.^19^

#### GBD Mortality, Incidence, and Prevalence Data

We extracted estimates of cause-specific prevalence and mortality by US state from the GBD 2021 study.^14,15^ These GBD estimates were produced using data from vital registration systems, censuses, household surveys, disease-specific registries, claims data, and other sources. See GBD 2021 Causes of Death Collaborators and GBD 2021 Diseases and Injuries Collaborators for detailed methods on these processes.^14,15^ The GBD 2021 input data sources and the state-specific prevalence and mortality estimates we extracted for this study are also available for download at the Global Health Data Exchange.^20^

### Step 2: Estimating Disease Prevalence for Each US County

There were 88 health conditions commonly defined and estimated in all 3 data sources. For these health conditions, we estimated county-level, age-specific, and sex-specific prevalence rates by regressing prevalence rates on mortality rates and a broad set of covariates at the state level using lasso regression. Lasso was used as it incorporates model selection into the model fitting process such that for each health condition a different set of covariates was included. After fitting our model using state-level data from the GBD study, we used county-level mortality from USHD and county-level covariate estimates to estimate county-level prevalence estimates. We did not estimate prevalence for 10 of the 88 health conditions because the linear regression used to estimate prevalence had an *R^2^* value of less than 0.9 for those 10 health conditions.

### Step 3: Explaining Variation in Cross-County Variation in Health Care Spending

We used the coefficient of variation to quantify relative variation in spending, computed across and within states. We used 2 types of decomposition analysis and linear regression to assess the factors contributing to cross-county variation in health care spending. We used Shapley decomposition to estimate the fraction of cross-county health care spending variation explained by each of the 4 factors. We used linear regression to assess variation in service utilization rates and service price and intensity rates. For each type of care, we separately regressed county-level, age-and-sex–specific, health condition–specific, and payer-specific utilization rates on median household income, insurance coverage, physicians per capita, and other available and relevant county-level covariates, controlling for differences in age and sex, health condition, and payer. We included separate intercepts for all combinations of age and sex, health condition, and payer to focus our analysis on variation across counties while fully controlling for age, sex, health condition, and payer. We separately repeated this process for service price and intensity. Lastly, we used Das Gupta decomposition to estimate for each state the factors driving differences between state-specific spending rates and the national average spending rates. Das Gupta decomposition is a method commonly used in demographic studies that assesses the difference between 2 rates and quantifies how each key factor contributes to that difference. In this analysis, the Das Gupta decomposition compares spending rates in each state to the national spending rates.

To generate an uncertainty interval, we used 50 independent, random draws of each estimate from the Disease Expenditure Project, USHD study, and GBD 2021 study. The analyses were applied to each draw independently, which propagated uncertainty through every stage of the process. To account for parameter uncertainty, we extracted estimates of the standard error. Data uncertainty and parameter uncertainty were combined using Rubin Rules.^21^ Analyses were completed with Python, version 3.11.8 (Python Software Foundation), and R software, version 4.4.0 (R Project for Statistical Computing). Two-sided significance testing and a *P*-value threshold of less than .05 were used to evaluate differences for statistical significance. The data analysis was conducted between March 2024 and July 2024.

## Results

**Figure 1.**
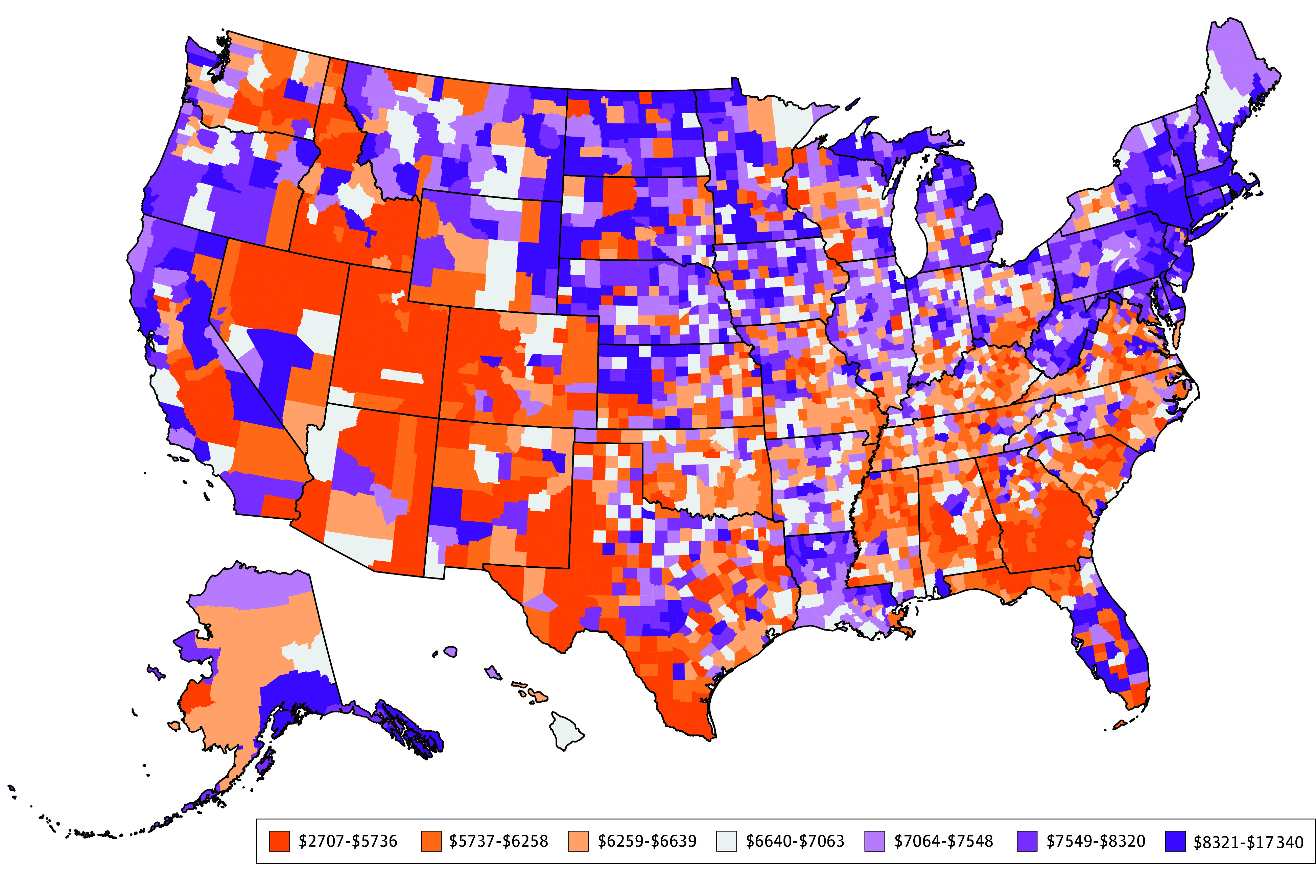
Health Care Spending Per Capita Across US Counties Spending is inclusive of all personal health care spending, excluding spending on durable medical equipment and over-the-counter drugs, and from Veteran Affairs, Department of Defense, and Indian Health Services.

In 2019, 76.6% of personal health care spending was included in this study. For 3110 US counties in 2019, health care spending per capita varied from $2707 in Chattahoochee County, GA, to $17 340 in Sumter County, FL (Figure 1). Spending was lowest in the Southwest and Southeast regions, and highest in the Northeast and Northern Plains, although in all regions and most US states there was considerable variation across counties. The between-state coefficient of variation was 0.14, while the mean within-state coefficient of variation was 0.15, meaning that within states there was generally as much variation as across states.

**Figure 2.**
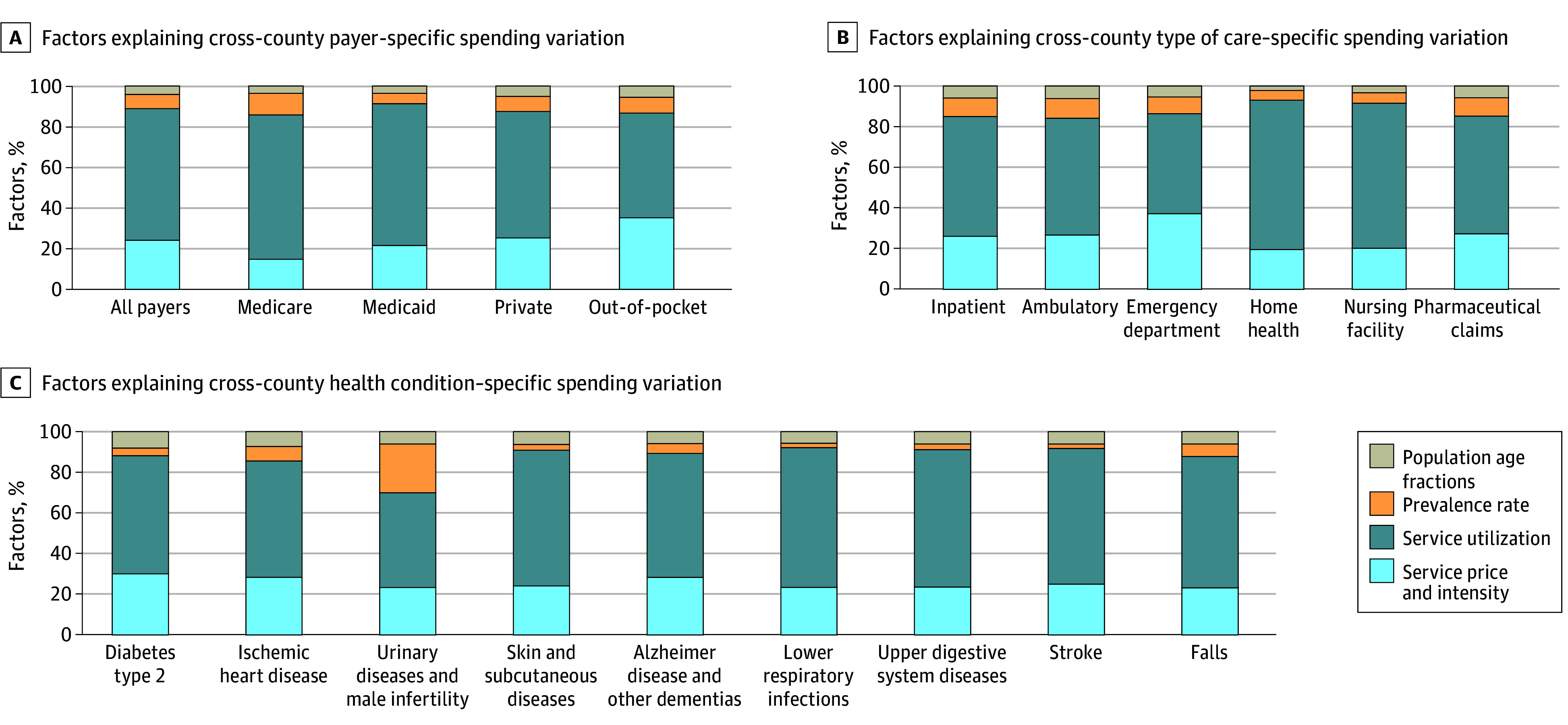
Factors That Explain Cross-County Variation in Spending Per Capita Factors explaining variation in total spending and by payer (A), type of care (B), and 9 highest-spending health conditions (C). Each bar shows the total variation explained by each factor. Because all variation was explained these percentages add up to 100%.

Overall, 64.8% (95% uncertainty interval [UI], 64.4%-65.1%) of cross-county variation was explained by variation in service utilization, 24.1% (95% UI, 23.8%-24.5%) was explained by service price and intensity, while disease prevalence and population age profile explained only 7.0% (95% UI, 6.9%-7.1%) and 4.1% (95% UI, 4.0%-4.2%) (Figure 2). These rates varied by payer; for private insurance and out-of-pocket spending, more variation in spending was attributed to service price and intensity compared to other payers. In contrast, variation in Medicare spending was almost entirely due to differences in utilization. These rates also varied by health condition.

**Figure 3.**
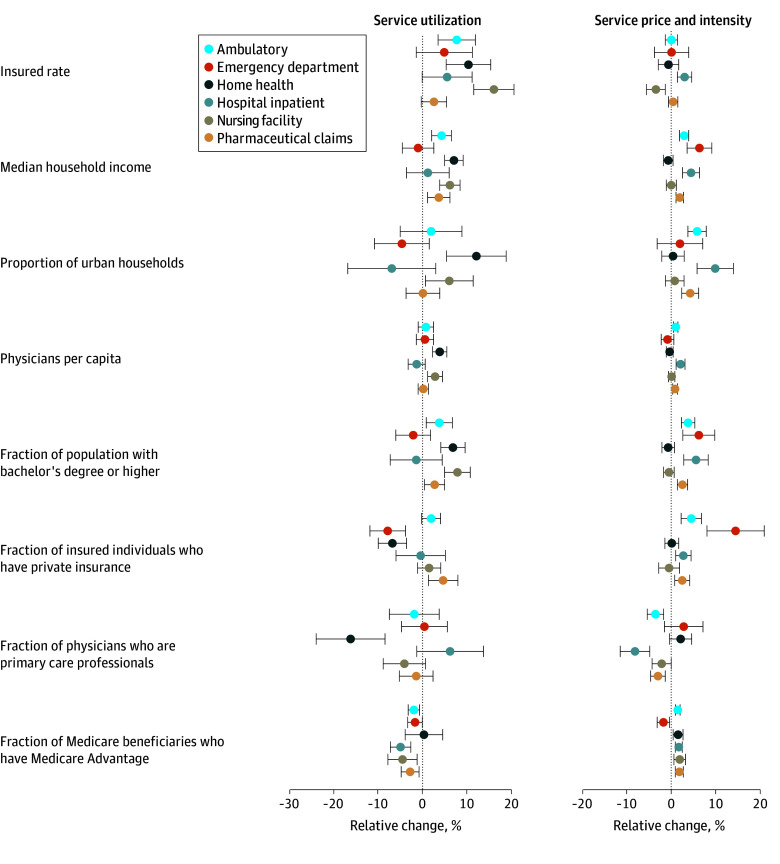
Factors Associated With Cross-County Service Utilization and Service Price and Intensity The estimated coefficient reports the relative change in service utilization or service price and intensity relative to an associated increase from the median to the 75th percentile of each independent variable. For example, an increase in health insurance rate from the median to the 75th percentile was associated with a 7.7% increase in ambulatory care utilization. Each bivariate regression was type-of-care-specific, assessing the relationship between type-of-care-specific service utilization and service price and intensity with county characteristics. Standard errors are clustered by payer, cause, and age and sex group and were adjusted for multiple hypothesis testing using the Bonferroni adjustment. This analysis considered 78 health conditions composing 55% of all health care spending.

Across counties, service utilization was associated with insurance coverage, median household income, and education (Figure 3). An increase from the median insurance coverage rate to the 75th percentile (a 3–percentage point difference) was associated with a 7.8% (95% UI, 3.6%-12.0%) increase in ambulatory care utilization. Median household income was associated with more utilization for all types of care except emergency department care and hospital inpatient utilization. The fraction of the population with a bachelor’s degree was associated with more utilization of nursing facilities, home health care, ambulatory care, and retail pharmaceuticals. The fraction of Medicare beneficiaries with Medicare Advantage was associated with less hospital inpatient, nursing facility, retail pharmaceutical, and ambulatory care utilization. An increase from the median Medicare Advantage coverage rate to the 75th percentile (an 8.6–percentage point difference) was associated with a 4.9% (95% UI, 2.6%-7.2%), 4.4% (95% UI, 1.1%-7.7%), 2.7% (95% UI, 0.7%-4.7%), and 1.9% (95% UI, 0.6%-3.2%), decrease in hospital inpatient, nursing facility, retail pharmaceutical, and ambulatory care utilization, respectively.

Many of the same factors that were associated with more service utilization were also associated with higher prices and intensity of care, including median income and education. The fraction of physicians who specialize in primary care was associated with lower prices and intensity of care, while Medicare Advantage was associated with higher prices and intensity of care.

**Figure 4.**
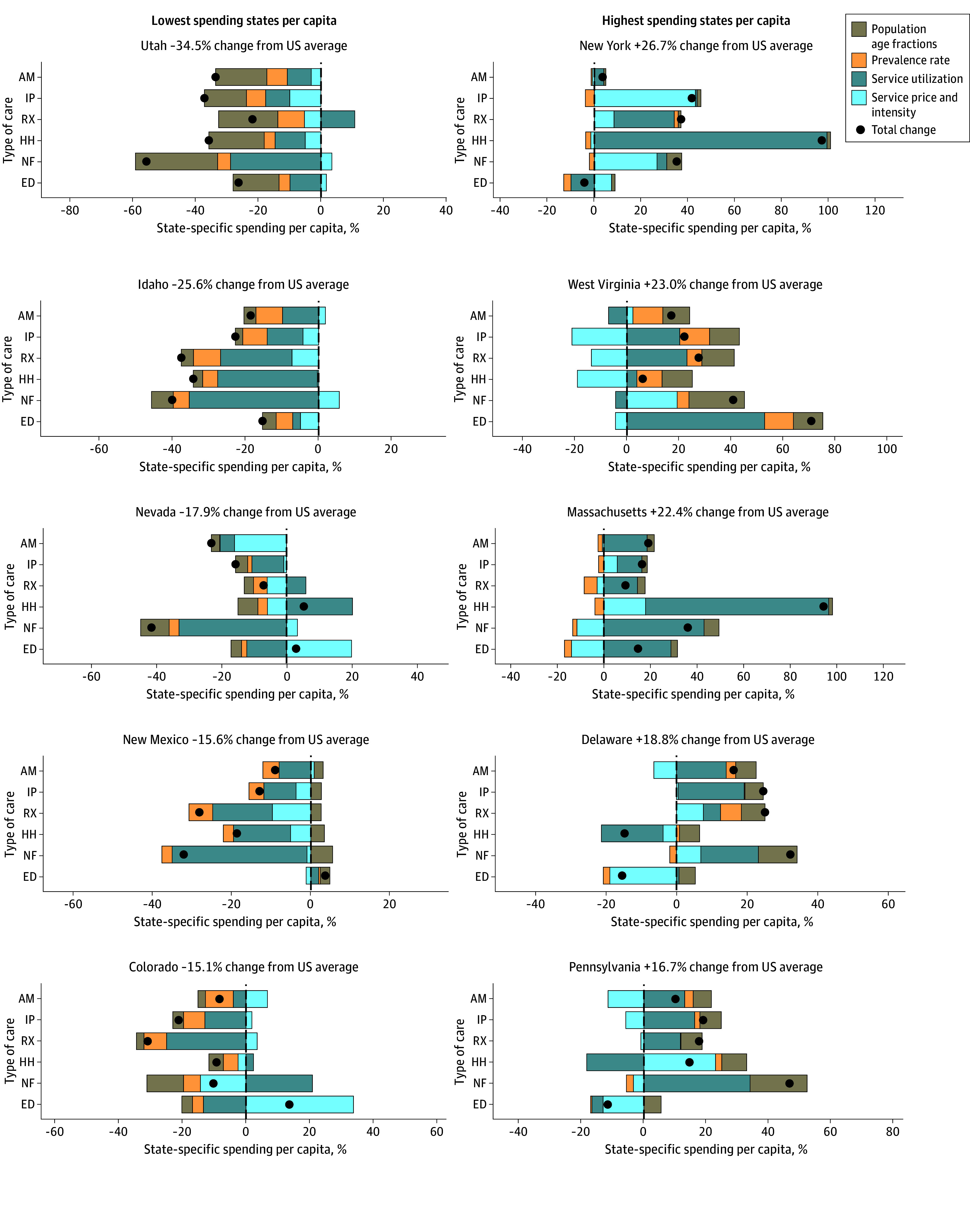
Factors Explaining State-Specific Spending Per Capita Relative to National Spending Per Capita by Type of Care for Highest- and Lowest-Spending States This analysis was completed including 78 health conditions that have disease prevalence estimated. The black dot is the difference between the state health care spending per capita estimate and the national health care spending per capita estimate for each type of care. The bars quantify the factors contributing to spending differences. For example, Utah spent 33.6% less on ambulatory care per capita than the national ambulatory care spending per capita level. All 4 factors contributed to this lower spending. AM indicates ambulatory care; ED, emergency department care; HH, home health; IP, inpatient care; NF, nursing facility care; and RX, retail prescribed pharmaceutical.

Figure 4 explains the drivers behind why each state spent, in per capita terms, more or less than the national average. Broadly, differences in service utilization drove a major portion of the differences in state-specific spending levels, followed by service price and intensity. While generally true, this finding varied across US states. Utah, the US state with the lowest per capita spending on health care in 2019, spent less on all types of care relative to the national mean. The age and sex distribution of the population contributed the most to this lower spending, although low rates of utilization for nursing facility care and emergency department care also played a role. Idaho spent the second least across all states, also spending less per capita for each of the types of care. The factor responsible for these lower-than-average spending rates was low service utilization. For New York, the state with the highest per capita health care spending, spending rates were highest relative to the national mean for hospital inpatient care, prescribed pharmaceuticals, and nursing facility care. For all 3 of these types of care, service price and intensity contributed substantially to the above-average spending amounts.

## Discussion

In this cross-sectional study, personal health care spending in the US varied dramatically among counties in 2019, with a more than 5-fold difference between the highest and lowest spending rates per capita. This variation was driven by a combination of factors. Service utilization explained a majority of the variation in spending per capita, while service price and intensity explained approximately one-fifth of the variation. Variations in population age profile and disease prevalence each explained a relatively small fraction of cross-county spending variation.

Several key factors were shown to be associated with service utilization and service price and intensity. Insurance coverage was associated with more utilization of ambulatory, home health, and nursing facility care. This finding reinforces previous research showing that while increasing insurance coverage may improve some self-reported health outcomes, it is not alone, in the short-run, a viable method for cost containment because it leads to more utilization.^22^ For health systems, which are charged with both ensuring access to essential services and sustaining health system financing, there is a necessary policy compromise. States seem to have come to divergent conclusions when considering this trade-off, as the uninsured rate ranges from 2% in Massachusetts, where spending is especially high, to 17% in Texas in 2022.

Median household income was found to be associated with greater utilization for all types of care except emergency department and hospital inpatient care. Furthermore, median income was associated with an increase in service price and intensity for ambulatory care, emergency department care, and hospital inpatient care. While outside of the control of the health system, low income is a critical social determinant of health, associated with worse health and lower health insurance coverage.^23^ Taken together these findings suggest a concerning inequity: the counties with the worst health, lowest insurance rates, and lowest incomes are receiving the least amount of health care.

The fraction of Medicare beneficiaries with Medicare Advantage was associated with less ambulatory care, hospital inpatient care, nursing facility care, and retail pharmaceutical utilization, while it was also associated with higher price and intensity for many of the same types of care. Health insurance market shifts toward value-based purchasing have been shown to be a means to driving down unnecessary utilization, but may also be associated with higher spending per visit, admission, and prescription.

Similar research to this present study showed that changes in service price and intensity were responsible for 50% of the increases in national spending over time between 1996 and 2013, while our study found that service price and intensity explained only 24.1% of the variation in spending per capita across counties in 2019.^24^ This nuanced juxtaposition suggests while prices and increased intensity of care are driving up spending over time, they do not explain a large fraction of the cross-county differences in spending levels. Service price and intensity of care were associated with greater out-of-pocket spending and private insurance spending. The finding that only 14.7% of the variation in Medicare spending was attributed to variation in price and intensity reflects the fact that Medicare prices are set at the national level with only small levels of variation permitted at the local level.

Our study should not be misinterpreted to suggest that variation in spending and utilization levels is bad. To the contrary, we show that disease prevalence and age of a county contribute to differences in spending. States like Utah and Florida stand out for relatively extreme spending levels (low and high, respectively), but given that these are the youngest and oldest states (and that spending increases with age), it is clear that neglecting to consider the age of the population results in a distorted perspective when evaluating the spending and comparing across states and time.

Our study does show that there is a great deal of unexplained variation in service utilization across all types of care. Critically, this research does not and cannot identify the right amount of utilization. Health outcomes vary dramatically across US counties and more research could focus on if there are optimal rates of utilization and/or price and intensity of care that are associated with better outcomes for certain populations.

Our study has implications for policymakers, payers, and physicians seeking to use variations in utilization and price and intensity as signals for where cost-containment strategies might be viable. First, our findings show that many states are spending more than would be expected based on their populations’ health care needs (ie, age and disease prevalence). Value-based payment models could be one means to identify specific diseases or places of care where the disconnect between burden and spending is highest. This research highlights this as Medicare Advantage coverage was shown to be associated with less service utilization. Second, our study’s finding that private insurance spending has almost twice as much spending variation attributed to price and intensity of care highlights the positive impact that price-reduction strategies could have on the private insurance market. Price-reduction strategies, such as national drug price negotiations, state-level reviews and regulation of insurance companies to implement caps for high-cost/low-value services, and standardization of billing and coding to minimize administrative complexity and overhead could help diminish the role that prices play in driving cross-county spending variation.

### Limitations

This study has several limitations, largely related to the input data used to produce the estimates. The input data for this study were statistically modeled to fill data gaps and address data biases. Those methods were peer-reviewed elsewhere, but inherent in those studies is uncertainty. In the present study, we have attempted to quantify that uncertainty and include it in the uncertainty reported in our own estimates using 50 draws of the input data that capture potential variation, although 50 draws are very few, and more draws may have led to different uncertainty intervals. In addition to data uncertainty, the analysis used to assess factors associated with utilization and price and intensity (Figure 3) incorporated parameter uncertainty and was adjusted for multiple hypothesis testing using the Bonferroni adjustment. Similar methods for quantifying parameter or model uncertainty were not available for the decomposition methods (Figure 2 and Figure 4). An additional limitation is that pharmaceutical spending estimates excluded pharmaceutical rebates and discounts. We did not control for local price level, even though prices for common goods as well as medical goods are known to vary across the US, because county-level price indices do not exist. Additionally, due to data limitations, we could not disaggregate service price and intensity into their individual components of service price, intensity, and technology. For the estimates produced for Figure 2, Figure 3, and Figure 4, only 78 health conditions were used. While these health conditions make up 55% of health spending, they may not reflect spending caused by other health conditions. The study period only extends through 2019 because this was the latest year of estimates available from several of our sources. Patterns in health care spending undoubtedly shifted dramatically during the COVID-19 pandemic (beginning in 2020); these shifts and the drivers that caused them are not captured in this study but will be the focus of future updates to the Disease Expenditure Project. Finally, this study was not designed nor able to identify excess or unnecessary service utilization or intensity of care, nor was the study causal; the estimated effects are merely associations.

## Conclusions

In this cross-sectional study, variation in health care spending among US counties was largely related to increases in health service utilization. Understanding the drivers of spending variation in the US can help policymakers assess the allocation of health care resources.

## Notes

Supplement 1. eAppendix. Assessing Drivers of Variation in Health Care Spending Across US Counties in 2019

Supplement 2. Data Sharing Statement
