# Error in Supplement 1

**DOI:** 10.1001/jamahealthforum.2025.0650

**Published:** 2025-04-04

The Original Investigation titled "Drivers of Variation in Health Care Spending Across US Counties,”^1^ published online on February 14, 2025, was corrected to fix Figures 5.3.1, 5.3.2, and 5.3.3 in the eAppendix in Supplement 1. The figures did not appear correctly in the original Supplement 1. This article has been corrected online.
