# Supplementary material for: Drivers of Variation in Health Care Spending Across US Counties
Source: JAMA Health Forum. 2025 Feb 14;6(2):e245220. doi: 10.1001/jamahealthforum.2024.5220 (PMC11829242; doi:10.1001/jamahealthforum.2024.5220)
Supplement: Supplement 1. — eAppendix. Assessing Drivers of Variation in Health Care Spending Across US Counties in 2019 [file jamahealthforum-e245220-s001.pdf]

Supplemental Online Content

Dieleman JL, Weil M, Beauchamp M, et al. Drivers of variation in health care spending across US counties. JAMA Health Forum. 2025;6(2):e245220.
doi:10.1001/jamahealthforum.2024.5220

eAppendix. Assessing Drivers of Variation in Health Care Spending Across US Counties in 2019

This supplemental material has been provided by the authors to give readers additional information about their work.

© 2025 Dieleman JL et al. JAMA Health Forum.

S1 Decomposition project framework 3
S1.1 Overview 3
S1.2 Appendix structure 3
S2 Sources of data 3
S2.1 DEX outputs 3
S2.2 Prevalence and incidence estimates 4
S2.3 Covariates 10
S3 Shapley Decomposition 12
S3.1 Overview 12
S3.2 Methodology 13
S3.3 Additional results 14
S4 Regression Analysis 27
S4.1 Overview 27
S4.2 Methodology 27
S4.3 Additional results 28
S5 Das Gupta Decomposition 35
S5.1 Overview 35
S5.2 Methodology 35
S5.3 Additional results 36
S6 GATHER Compliance 49
References 54

© 2025 Dieleman JL et al. JAMA Health Forum.

S1 Decomposition project framework

S1.1 Overview

This project aimed to analyze drivers of variation in health care spending and utilization in the United States across geographical regions. It is an extension of previous research conducted by the Disease Expenditure (DEX) team at the Institute for Health Metrics and Evaluation, which produced county-level estimates of healthcare spending and utilization by health condition, payer, type of care setting, age, sex, and year.

S1.2 Appendix structure

In Section 1, we provide an overview of this project's framework and strategy. In Section 2, we summarize the various data sources used in this analysis and the processing steps for each. In Sections 3-5, we outline the statistical processes used in this paper, the basis and application of each, and any additional steps used to validate results.

S2 Sources of data

S2.1 DEX outputs

The estimates for spending, utilization, and population counts used in this research were produced by the DEX team at the Institute for Health Metrics and Evaluation. Using over 64 billion insurance claims and over 800 million administrative records spanning from 2010-2019, the DEX researchers assigned each claim or encounter one of 38 age-sex groups, one of seven types of care (ambulatory, inpatient, pharmaceutical, nursing facility, dental, home health, and emergency department), and one of four payer categories (private insurance, Medicare, Medicaid, and out-of-pocket). That analysis excluded data on spending on durable and non-durable medical equipment (including over-the-counter drugs and medical products such as bandages), medical transportation, and non-personal health care (including research and development and public health) and spending from Veteran Affairs, Tri-care, and Indian Health Services. Each health system encounter reported in the data was assigned to one of 148 health conditions. Records were adjusted for inconsistencies in the data, spending was reallocated from primary health condition to key comorbidities using regression analyses, and a small area model was used to estimate spending in low-population areas where data were insufficient. Estimates were scaled to align with official State Health Expenditure Accounts. In the analysis presented here, only estimates for 2019 were used.

© 2025 Dieleman JL et al. JAMA Health Forum.

S2.2 Prevalence and incidence estimates

To capture the number of cases for each health condition we used either prevalence or incidence, depending on if the health condition was acute or chronic. We used incidence for all cancers, injuries, acute infections, and stroke, and used prevalence for all other health conditions. This distinction was made to align the definition of cases with whichever metric most dictates the need for treatment for each health condition. A list of all included health conditions and which metric was used is provided in Table 2.2.1.

The county-level estimates of prevalence and incidence were generated via linear regression from county-level mortality estimated by the United States Health Disparities (USHD) team at IHME and state-level prevalence and incidence estimated by the Global Burden of Disease project (GBD) at IHME. The USHD researchers applied small-area estimation models to estimate mortality rates. See GBD US Health Disparities Collaborators for detailed methods on these processes and for a complete list of US county mortality data sources.[11] GBD researchers modeled cause-specific incidence and prevalence by US state using a tool known as disease model—Bayesian meta-regression version 2.1 (DisMod-MR 2.1), while cause-specific mortality by US state was modelled using a tool known as cause of death ensemble modeling (CODEm).

To impute prevalence and incidence by health condition at the county level, we fit cause-specific LASSO regressions on the relationship between prevalence (or incidence) and mortality, accounting for age, sex, state, and covariate effects. We then used county-level mortality to predict prevalence (or incidence) at the county level and scaled predicted prevalence to aggregate to the GBD state prevalence.

There were 88 health conditions commonly defined across the three data sources, 78 of which were then used for this present analysis (Table 2.2.1). For these health conditions we estimated county-level, age and sex-specific prevalence rates by regressing prevalence rates on mortality rates and a broad set of covariates at the state-level using lasso regression. Lasso was used as it incorporates model selection into the model fitting process such that for each health condition a different set of covariates was included. The lasso regressions were fit using the *glmnet* package in *R*. We used the built in cross-validation function (*cv.glmnet)* to perform cross validation and selected the value of lambda that gave the most regularized model such that the cross-validated error was within one standard error of the minimum (*lambda.1se).* After fitting our model using state level data from the GBD, we used county-level mortality estimates from USHD to estimate county-level prevalence estimates. Within each state, the relative variation in county-level prevalence was forced to equal the relative variation in county-level mortality. This process was completed separately for each of the health conditions and for each sex. The equation

© 2025 Dieleman JL et al. JAMA Health Forum.

used for the LASSO regression is provided below where l = location, a = age group, and X is a set of covariates.

$$\ln(prevalence\ rate)_{la} \sim \alpha_l + \alpha_a + \beta \ln(mortality\ rate)_{la} + \gamma X_{la} + \varepsilon_{la}$$

Covariates included for possible selection by LASSO were: median household income, medical doctorates per capita, population density, unemployment rate, tobacco source availability, proportion of the population with a bachelor's degree, proportion of the population with a high school degree, poverty rate, proportion of population on reservation land, proportion of Hispanic, proportion non-Hispanic black, proportion non-Hispanic American Indian or Alaskan Native, proportion non-Hispanic Asian/Pacific Islander, proportion non-Hispanic white, and proportion with other race.

After fitting the LASSO regressions, health conditions were included in the analysis if 1) mortality was selected and had a positive coefficient and 2) the R2 on both sex-specific regressions was greater than 0.90. For sex-restricted health conditions such as cervical cancer, the second condition only applied to the relevant sex. After applying these inclusion thresholds, the health condition set was reduced from 88 to 78.

Table 2.2.1 List of included health conditions

Health Condition	Case Definition
**Enteric infections**	prevalence
**HIV/AIDS**	prevalence
**Neonatal preterm birth**	prevalence
**Nutritional deficiencies**	prevalence
**Meningitis**	prevalence
**Lower respiratory infections**	prevalence
**Tuberculosis**	prevalence
**Interpersonal violence**	incidence
**Self-harm**	incidence
**Transport injuries**	incidence
**Other unintentional injuries**	incidence
**Falls**	incidence
**Atrial fibrillation and flutter**	prevalence
**Cardiomyopathy and myocarditis**	prevalence
**Endocarditis**	prevalence
**Hypertensive heart disease**	prevalence
**Ischemic heart disease**	prevalence
**Other cardiovascular and circulatory diseases**	prevalence
**Lower extremity peripheral arterial disease**	prevalence
**Rheumatic heart disease**	prevalence

© 2025 Dieleman JL et al. JAMA Health Forum.

**Stroke**	prevalence
**Non-rheumatic valvular heart disease**	prevalence
**Chronic obstructive pulmonary disease**	prevalence
**Interstitial lung disease and pulmonary sarcoidosis**	prevalence
**Chronic kidney disease**	prevalence
**Diabetes mellitus type 1**	prevalence
**Diabetes mellitus type 2**	prevalence
**Cirrhosis and other chronic liver diseases**	prevalence
**Gallbladder and biliary diseases**	prevalence
**Inguinal, femoral, and abdominal hernia**	prevalence
**Inflammatory bowel disease**	prevalence
**Pancreatitis**	prevalence
**Upper digestive system diseases**	prevalence
**Vascular intestinal disorders**	prevalence
**Rheumatoid arthritis**	prevalence
**Bladder cancer**	incidence
**Malignant neoplasm of bone and articular cartilage**	incidence
**Brain and central nervous system cancer**	incidence
**Breast cancer**	incidence
**Cervical cancer**	incidence
**Colon and rectum cancer**	incidence
**Esophageal cancer**	incidence
**Gallbladder and biliary tract cancer**	incidence
**Hodgkin lymphoma**	incidence
**Kidney cancer**	incidence
**Larynx cancer**	incidence
**Leukemia**	incidence
**Liver cancer**	incidence
**Tracheal, bronchus, and lung cancer**	incidence
**Non-Hodgkin lymphoma**	incidence
**Malignant skin melanoma**	incidence
**Mesothelioma**	incidence
**Lip and oral cavity cancer**	incidence
**Multiple myeloma**	incidence
**Nasopharynx cancer**	incidence
**Non-melanoma skin cancer**	incidence
**Other neoplasms**	incidence
**Other malignant neoplasms**	incidence
**Other pharynx cancer**	incidence
**Ovarian cancer**	incidence
**Pancreatic cancer**	incidence
**Prostate cancer**	incidence

© 2025 Dieleman JL et al. JAMA Health Forum.

**Stomach cancer**	incidence
**Thyroid cancer**	incidence
**Soft tissue and other extraosseous sarcomas**	incidence
**Uterine cancer**	incidence
**Alzheimer's disease and other dementias**	prevalence
**Idiopathic epilepsy**	prevalence
**Multiple sclerosis**	prevalence
**Motor neuron disease**	prevalence
**Other neurological disorders**	prevalence
**Parkinson's disease**	prevalence
**Congenital birth defects**	prevalence
**Endocrine, metabolic, blood, and immune disorders**	prevalence
**Urinary diseases and male infertility**	prevalence
**Skin and subcutaneous diseases**	prevalence
**Alcohol use disorders**	prevalence
**Opioid use disorders**	prevalence

Figure 2.2.1 Comparison of county prevalence estimates using LASSO and OLS

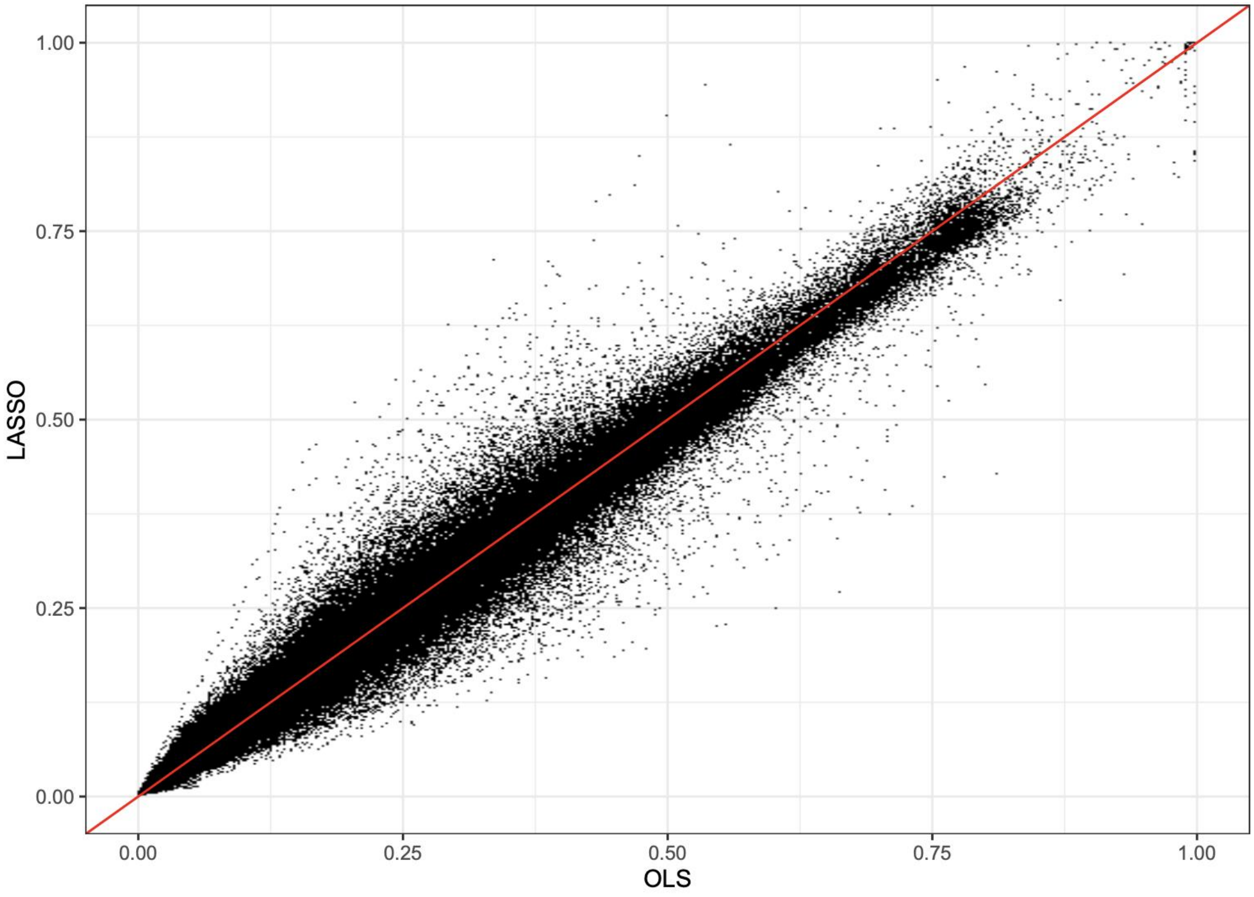

Figure 2.2.2 Relationship between disease prevalence and mortality

© 2025 Dieleman JL et al. JAMA Health Forum.

Figure 2.2.2 Relationship between disease prevalence and mortality

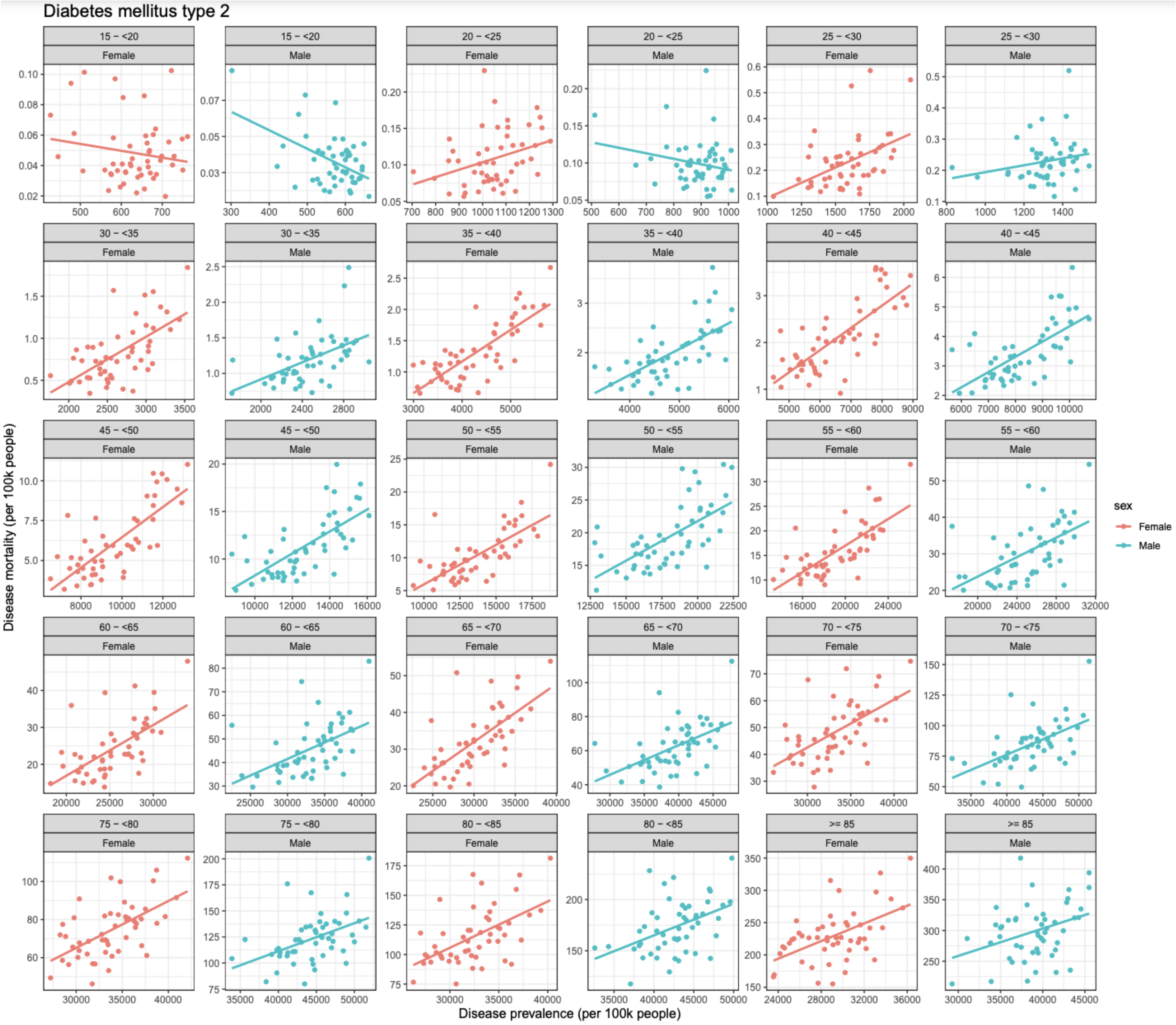

© 2025 Dieleman JL et al. JAMA Health Forum.

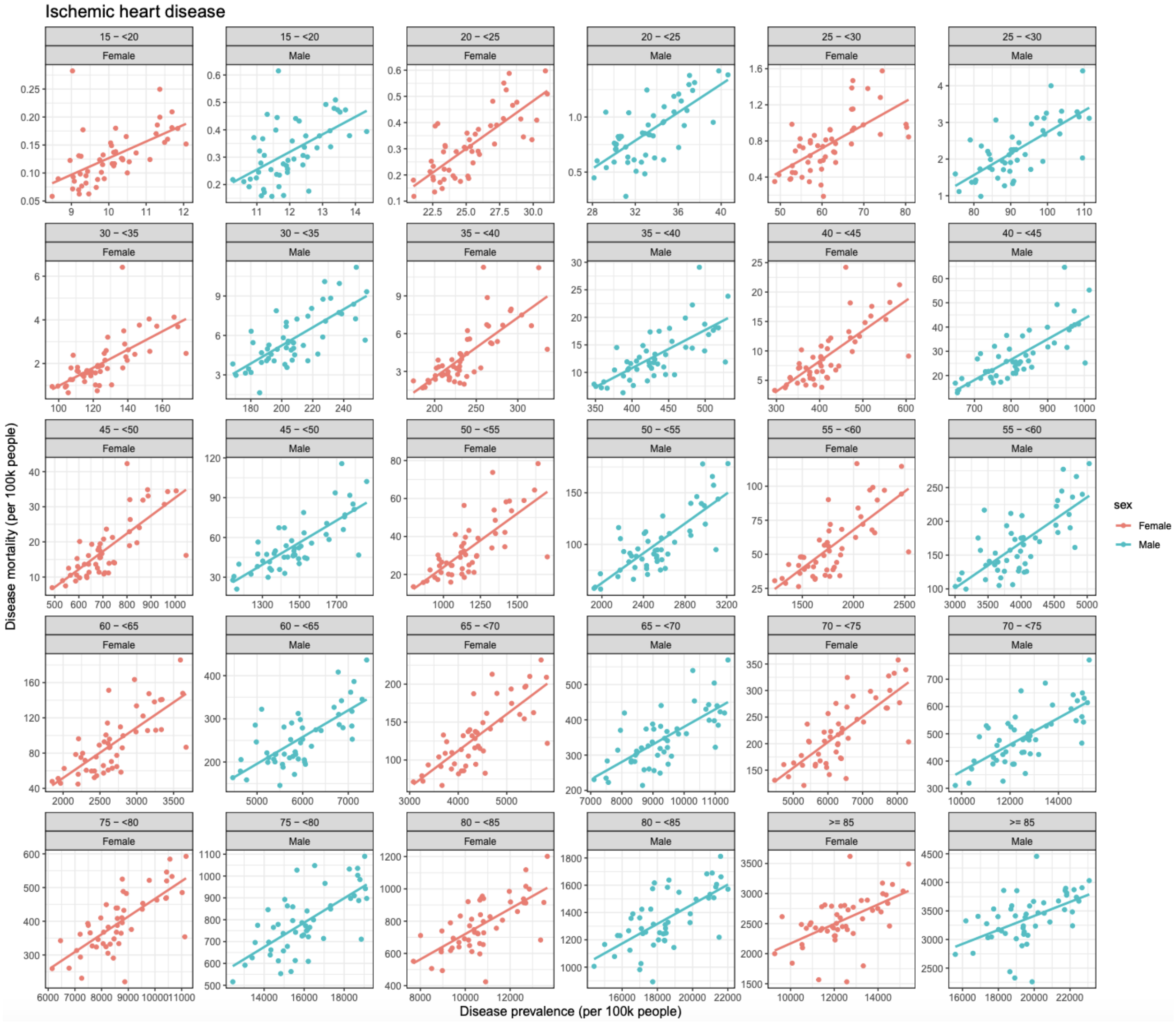

© 2025 Dieleman JL et al. JAMA Health Forum.

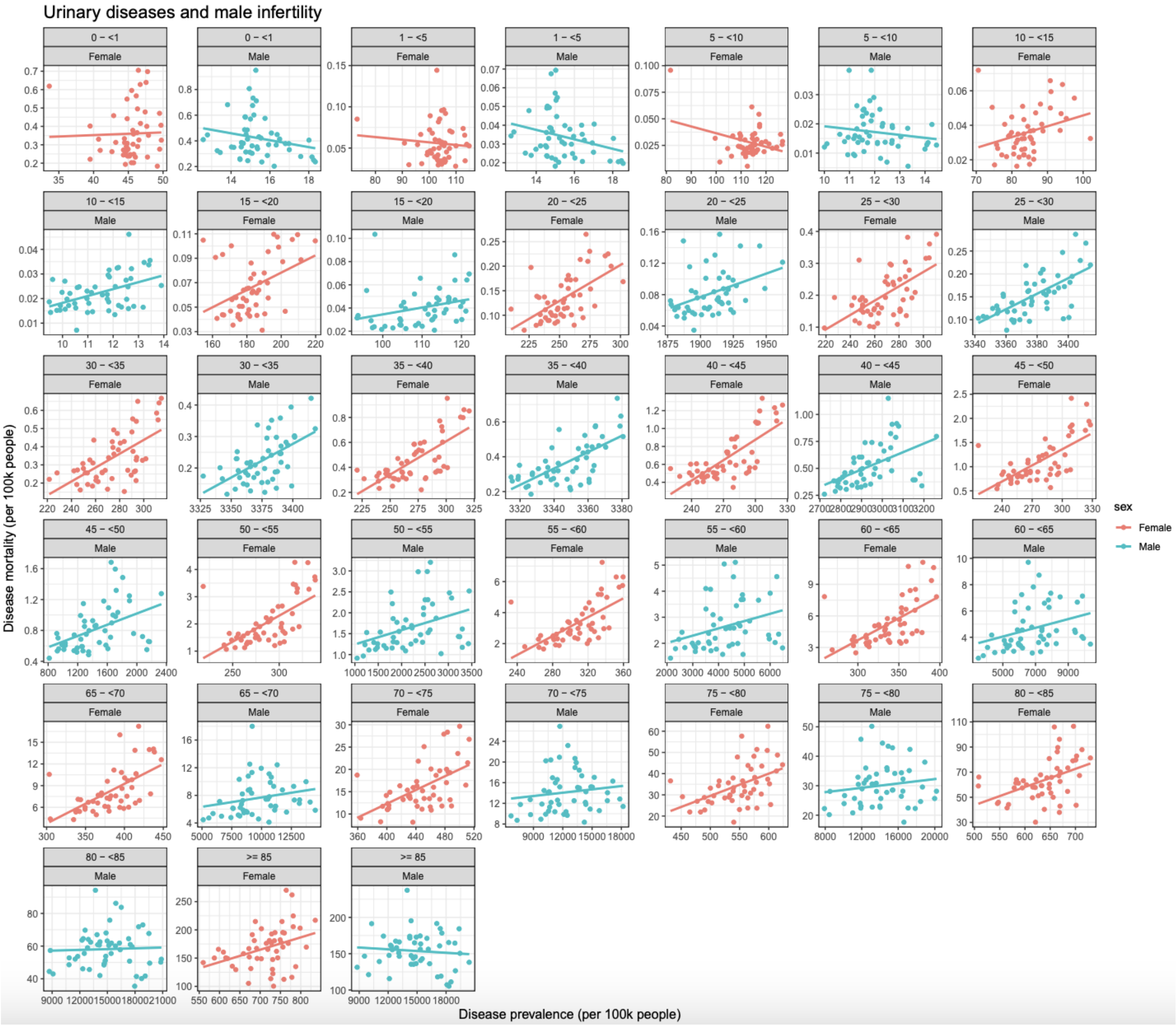

S2.3 Covariates

The estimates for insured rate, median household income, proportion of urban households, and number of medical doctorates (MDs) per capita, were estimated by the United States Health Disparities (USHD) team at the Institute for Health Metrics and Evaluation. These covariates are location specific to one of 3110 US counties and are not further disaggregated. Each covariate was estimated using data collected from a variety of sources, as listed in Table 2.3.1 below.

Table 2.3.1

Covariate Name	Covariate Definition	Source(s)
Insured rate	Number of individuals in county covered by some	US Census Bureau's Small Area Health Insurance Estimates Program

© 2025 Dieleman JL et al. JAMA Health Forum.

	form of health insurance per capita.	US Census Bureau's American Community Survey
Median household income	Median household income in county.	US Census Bureau<br><br>US Census Bureau's Small Area Income and Poverty Estimates Program<br><br>US Bureau of Labor Statistic's Local Area Unemployment Statistics
Proportion of urban households	Percent of households located in an urban setting within county.	US Census Bureau<br><br>US Department of Agriculture's Rural-Urban Continuum Codes
MDs per capita	Number of individuals who hold a medical doctorate within county per capita.	Health Resources & Services Administration's Area Health Resource Files
Bachelor's degree+ rate	Proportion of individuals within county that hold a bachelor's degree or above.	US Census Bureau<br><br>US Census Bureau's American Community Survey
Fraction of insured that are privately insured	Fraction of all insured individuals within county that are covered by private insurance.	US Census Bureau's Small Area Health Insurance Estimates Program<br><br>US Census Bureau's American Community Survey
Fraction of MDs that are Primary Care Providers	Fraction of individuals who hold a medical doctorate within county that are primary care providers.	Health Resources & Services Administration's Area Health Resource Files
Fraction of Medicare that is Medicare Advantage	Fraction of individuals who are covered by Medicare within a county that are covered by Medicare Advantage.	Centers for Medicare & Medicaid Services Enrollment Data

© 2025 Dieleman JL et al. JAMA Health Forum.

S3 Shapley Decomposition

S3.1 Overview

To quantify the fraction of total variation in spending attributable to each of four key drivers, we use the Shapley method to decompose the R-squared. These factors were (1) the proportion of the total population in a particular age-sex group, (2) health condition incidence (for all injuries and cancers) or prevalence (for all other diseases), (3) service utilization, and (4) service price and intensity (which reflects the level of spending per health care encounter), including the use of new technologies. For each age-sex-payer-type of care-condition-location combination we calculated each of the four factors as:

$$Age - Sex\ Population\ Proportion = \frac{Pop_{a,s,l}}{Pop_l}$$

$$Condition\ Incidence\ or\ Prevalence\ = \frac{Cases_{a,s,c,l}}{Pop_{a,s,l}}$$

$$Service\ Utilization = \frac{Encounters_{a,s,p,t,c,l}}{Cases_{a,s,c,l}}$$

$$Service\ Price\ and\ Intensity = \frac{Spending_{a,s,p,t,c,l}}{Encounters_{a,s,p,t,c,l}}$$

Where c indicates one of 79 health conditions, a and s indicate one of 38 age and sex groups, p indicates one of four payers, t indicates one of seven types of care, and l indicates one of 3110 US counties.

To create a linear relationship between the above variables and spending per capita, the natural log of health care spending per capita was defined as the sum of natural log of the above four factors using the following equation:

$$\ln\left(\frac{Spending_{a,s,\mathrm{p},\mathrm{t},c,l}}{Pop_l}\right) = \alpha_{a,s,p,t,c} + \beta_1 \ln\left(\frac{Pop_{a,s,l}}{Pop_l}\right) + \beta_2 \ln\left(\frac{Cases_{a,s,c,l}}{Pop_{a,s,l}}\right) + \beta_3 \ln\left(\frac{Encounters_{a,s,p,t,c,l}}{Cases_{a,s,c,l}}\right) + \beta_4 \ln\left(\frac{Spending_{a,s,p,t,c,l}}{Encounters_{a,s,p,t,c,l}}\right) + \varepsilon_{a,s,p,t,c,l}$$

For ambulatory, dental, emergency department care, and home health care, one encounter was measured as one visit, so we calculated service utilization as the mean number of visits per prevalent (or incident) case and service price and intensity as mean spending per visit. For hospital inpatient and nursing facility care, one encounter was

© 2025 Dieleman JL et al. JAMA Health Forum.

measured as one admission, so service utilization was calculated as the number of admissions per prevalent (or incident) case and price and intensity was calculated as the spending amount per admission. For retail pharmaceuticals, one encounter was measured as purchased prescription, service utilization was calculated as the mean number of purchased prescriptions per prevalent cases and service price and intensity as mean spending per purchased prescription.

The above four factors were estimated for ~55K unique age-sex-payer-type of care-condition combinations for each location in 2019. To estimate the relative effect of each of the four factors on variance in spending per capita across 3110 counties, we conducted a decomposition analysis using the methods described by Shorrocks (2013).

S3.2 Methodology

To conduct Shapley decomposition, estimates of the Pop variable above were obtained from the USHD study, $Cases$ were taken from the USHD study and GBD 2021 and estimates of $Utilization$ and $Spending$ came from the Disease Expenditure 2019 study. Specific processing for the $Cases$ variable is detailed in Section 2.3 above, which was undertaken prior to Shapley decomposition.

The four factors were then calculated using the equations noted above for each combination of age-sex-payer-type of care-condition for all county locations 2019. This resulted in a total of ~170M observations across all locations.

To account for $\alpha_{a,s,p,t,c}$ we demeaned the dependent variable and each independent variable by the age, sex, payer, type of care, and cause specific mean across counties. This is mathematically equivalent to having ~55k unique intercepts.

The demeaned data was regressed using the linear model equation above. The R-squared value for this regression was then decomposed using the Shapley decomposition method in R using a package developed by Elbers (2024). Results were averaged across 50 draws of data. Each Shapley decomposition was run at several levels of granularity, show in Table 3.2.1 below.

Table 3.2.1

Faceted variable	Number of runs	Observations per run
**None**	1	~170M
**Payer**	4	~42M
**Type of care**	6	~28M
**Health condition**	79	~2M

© 2025 Dieleman JL et al. JAMA Health Forum.

S3.3 Additional results

To analyze the stability of our Shapley decomposition results across time, we repeated the methods above for two additional years of data, 2010 and 2015. These results are shown below in Figure 3.3.1 and Figure 3.3.2, respectively.

© 2025 Dieleman JL et al. JAMA Health Forum.

Figure 3.3.1

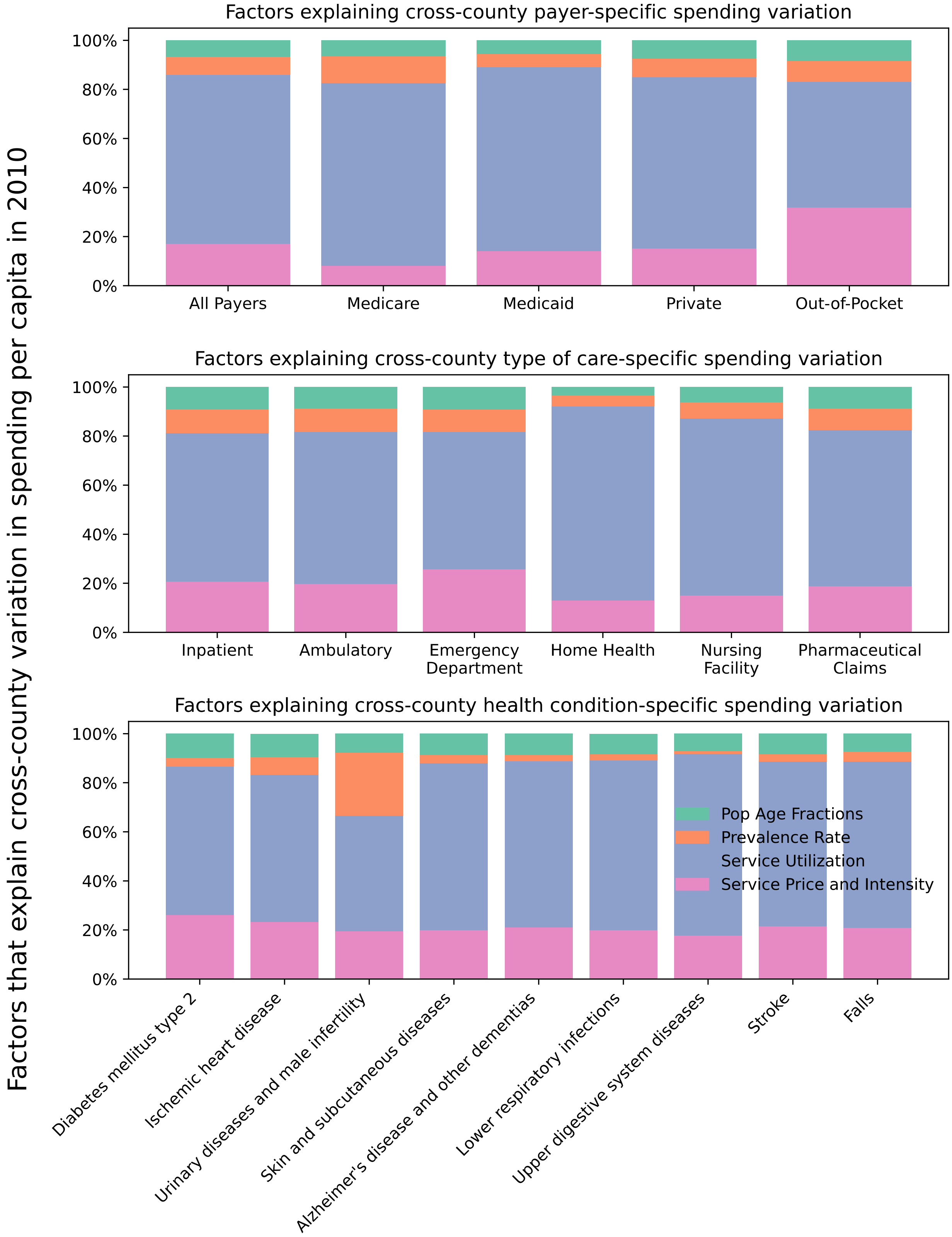

© 2025 Dieleman JL et al. JAMA Health Forum.

Figure 3.3.2

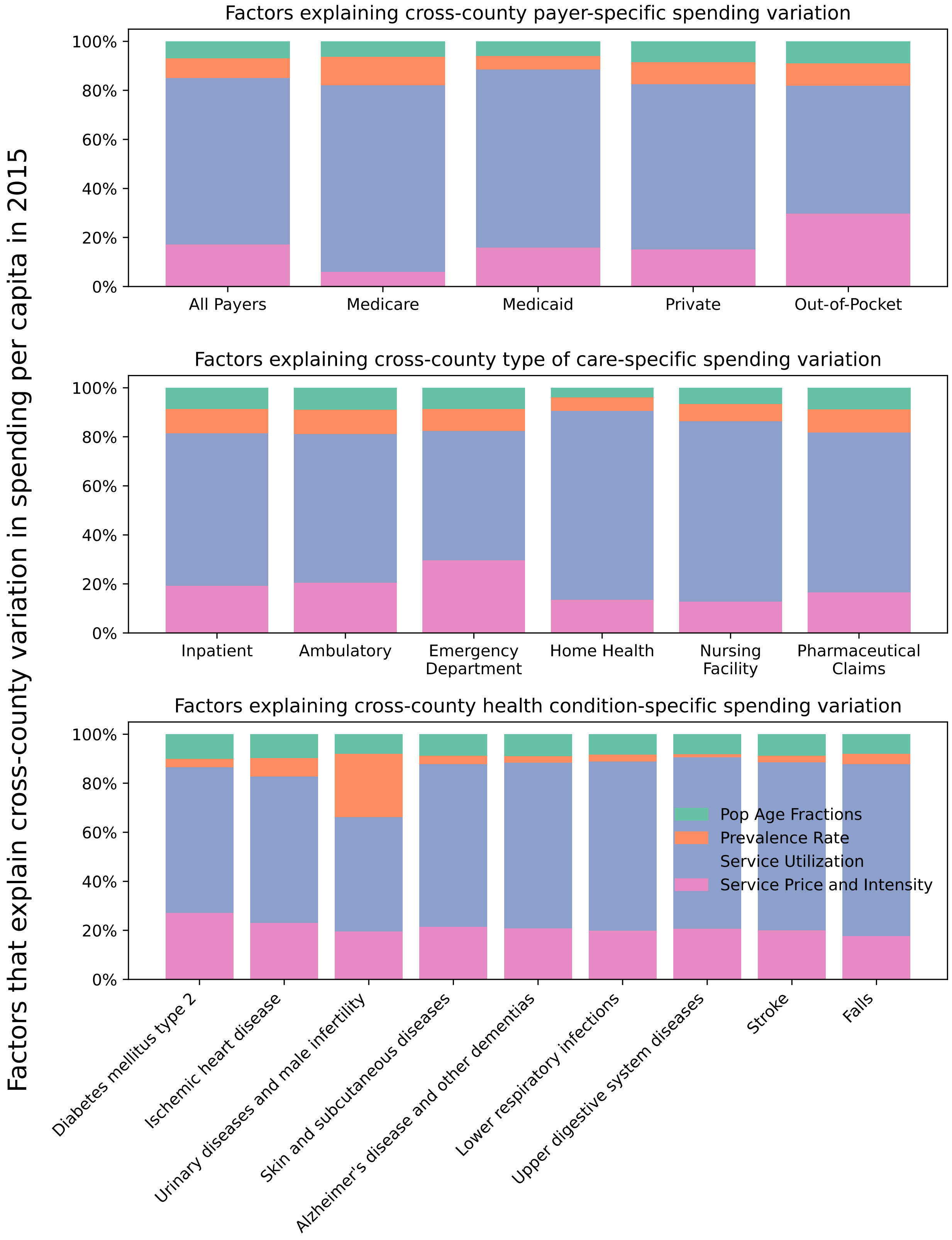

© 2025 Dieleman JL et al. JAMA Health Forum.

We also conducted a sensitivity analysis to explore the possibility that disease prevalence is higher for Medicaid-insured population than the privately insured population. In this scenario, all prevalence estimates for the Medicaid population were inflated by 20% and prevalence estimates in the privately insured population were deflated by 20%. The resulting payer-specific Shapley decomposition is shown below in Figure 3.3.3.

Figure 3.3.3

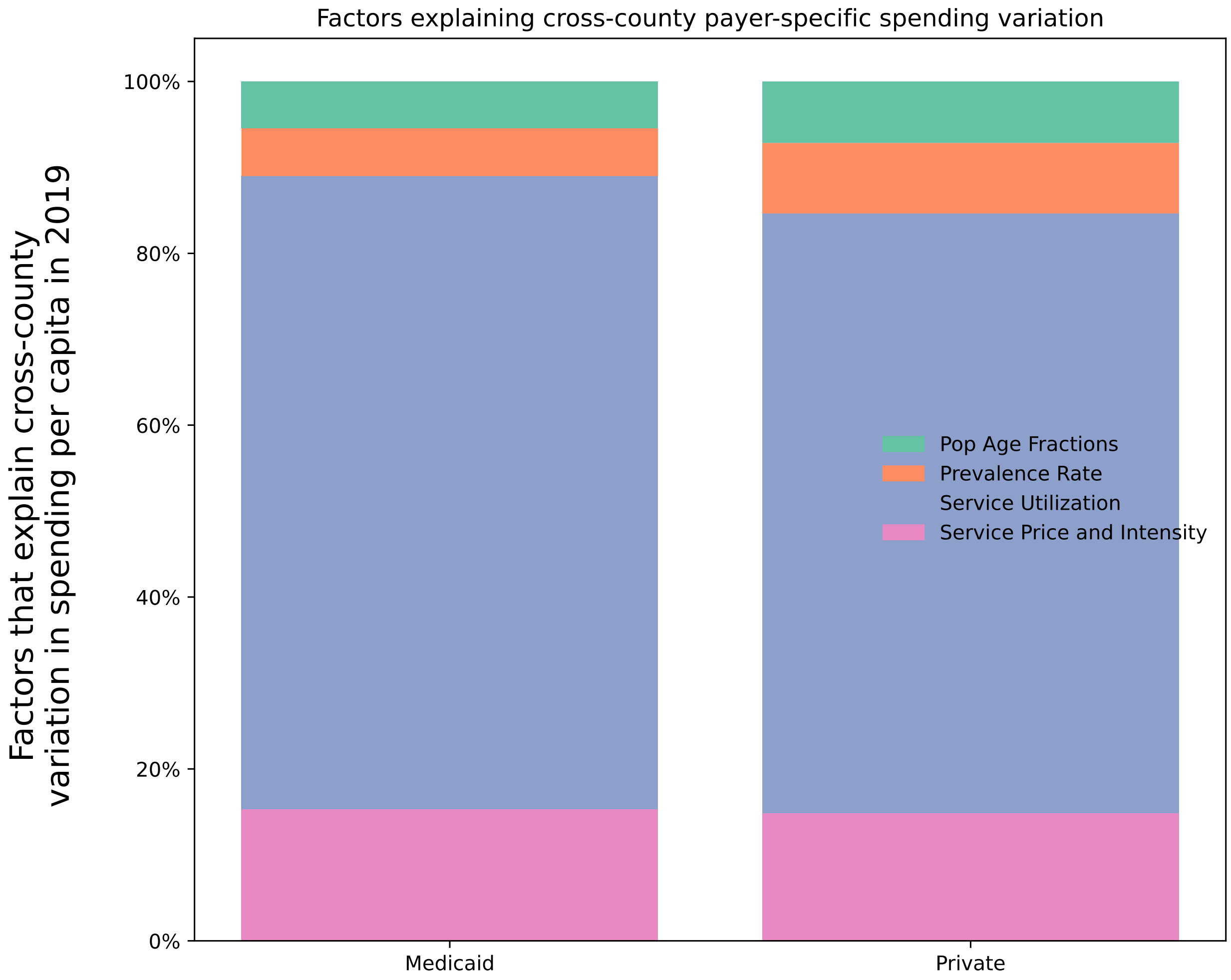

To validate the robustness of the overall Shapley decomposition run across ~170M observations, we performed an additional aggregate decomposition. This was performed by running Shapley decomposition on each combination of payer-type of care-condition independently, resulting in 1896 total runs. The Shapley values output from these runs were then aggregated using a weighted average, with weights based on the fraction of total spending that a stratum of data contributed to overall spending. The resulting aggregated Shapley values from this check are shown below in Figure 3.3.4.

© 2025 Dieleman JL et al. JAMA Health Forum.

Figure 3.3.4

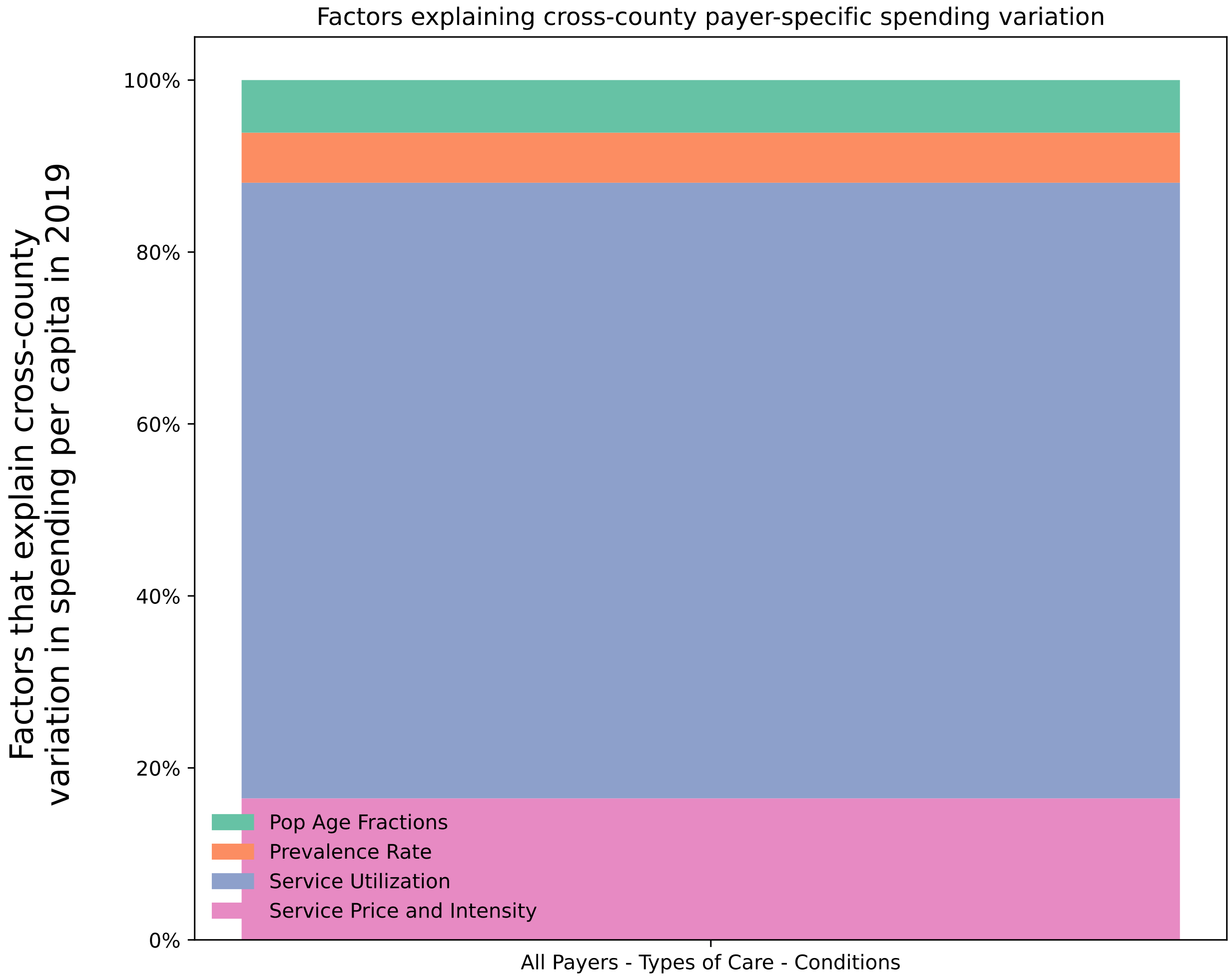

A version of Shapley decomposition was also created using mortality instead of prevalence. Mortality is generally better measured than prevalence and incidence because the estimates rely on vital registration systems and death certificates. The same steps were performed as noted above, but with the following definitions and equation:

$$Age - Sex\ Population\ Proportion = \frac{Pop_{a,s,l}}{Pop_l}$$

$$Condition\ Morality\ = \frac{Deaths_{a,s,c,l}}{Pop_{a,s,l}}$$

$$Service\ Utilization = \frac{Encounters_{a,s,p,t,c,l}}{Deaths_{a,s,c,l}}$$

$$Service\ Price\ and\ Intensity = \frac{Spending_{a,s,p,t,c,l}}{Encounters_{a,s,p,t,c,l}}$$

© 2025 Dieleman JL et al. JAMA Health Forum.

$$
\ln\left(\frac{Spending_{a,s,\mathrm{p},\mathrm{t},c,l}}{Pop_l}\right) = \alpha_{a,s,p,t,c} + \beta_1 \ln\left(\frac{Pop_{a,s,l}}{Pop_l}\right) + \beta_2 \ln\left(\frac{Deaths_{a,s,c,l}}{Pop_{a,s,l}}\right) + \beta_3 \ln\left(\frac{Encounters_{a,s,p,t,c,l}}{Deaths_{a,s,c,l}}\right) + \beta_4 \ln\left(\frac{Spending_{a,s,p,t,c,l}}{Encounters_{a,s,p,t,c,l}}\right) + \varepsilon_{a,s,p,t,c,l}
$$

Results for this analysis are shown in Figure 3.3.5 below.

© 2025 Dieleman JL et al. JAMA Health Forum.

Figure 3.3.5

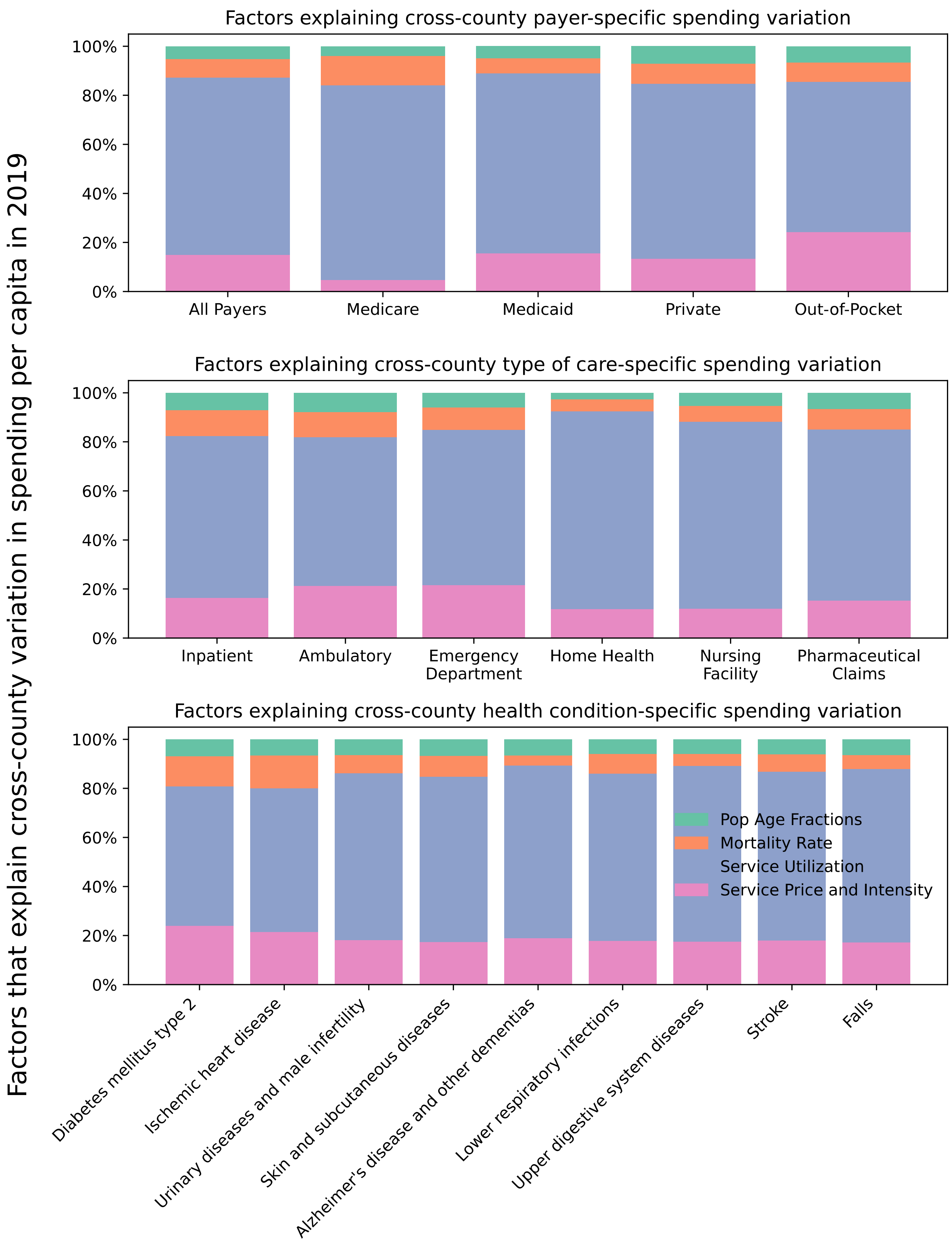

© 2025 Dieleman JL et al. JAMA Health Forum.

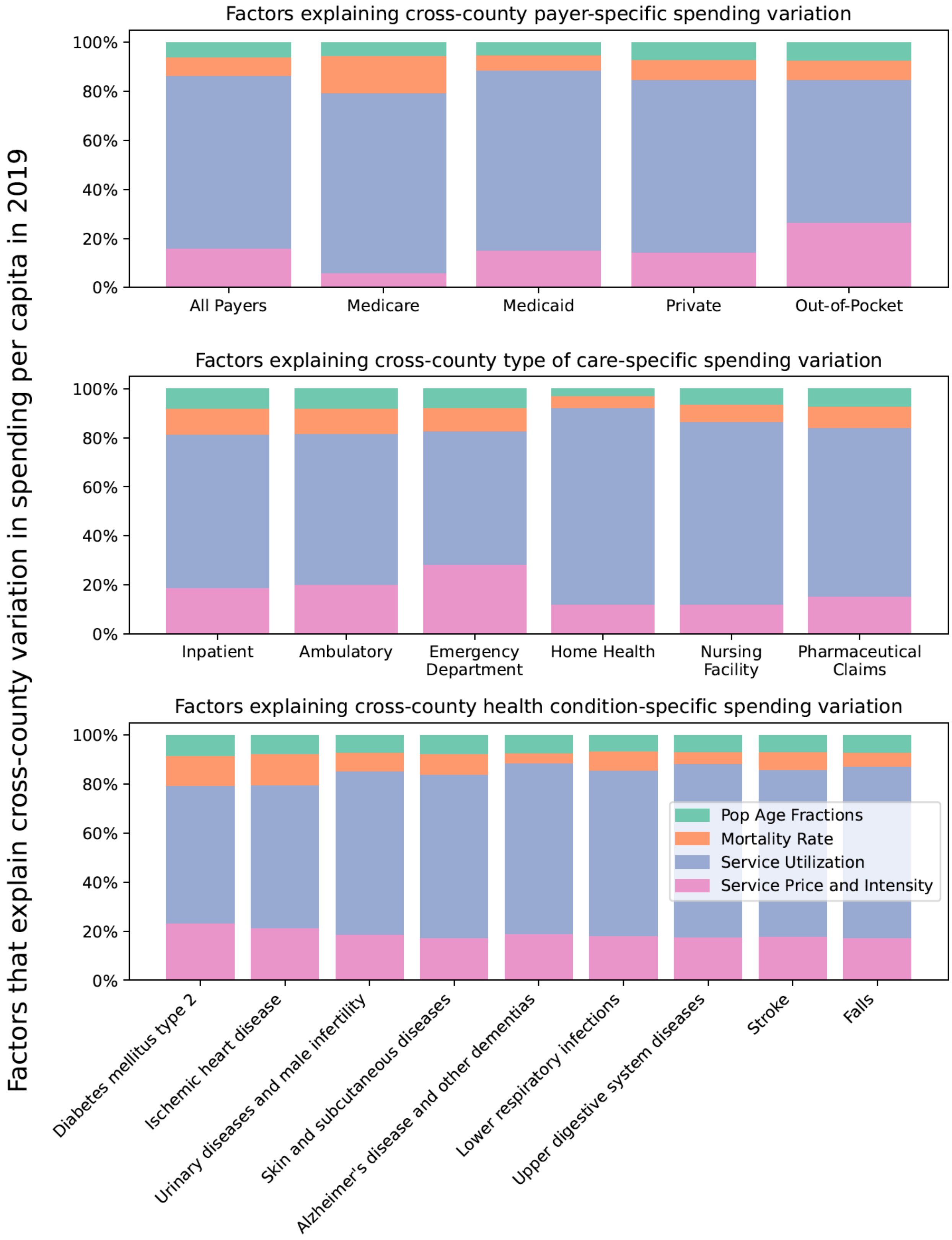

© 2025 Dieleman JL et al. JAMA Health Forum.

Shapley decomposition was also tested at state level to confirm the approximate contribution of each factor to county variation. This was accomplished by substituting the 3110 county locations used for 50 state locations. This resulted in a total of ~2.8M observations due to the increase in geographical granularity. The final dataset was regressed using all observations, rather than using all possible facets listed in Table 3.2.1. The Shapley decomposition of R-squared values at the state level is shown in Figure 3.3.6 below.

© 2025 Dieleman JL et al. JAMA Health Forum.

Figure 3.3.6

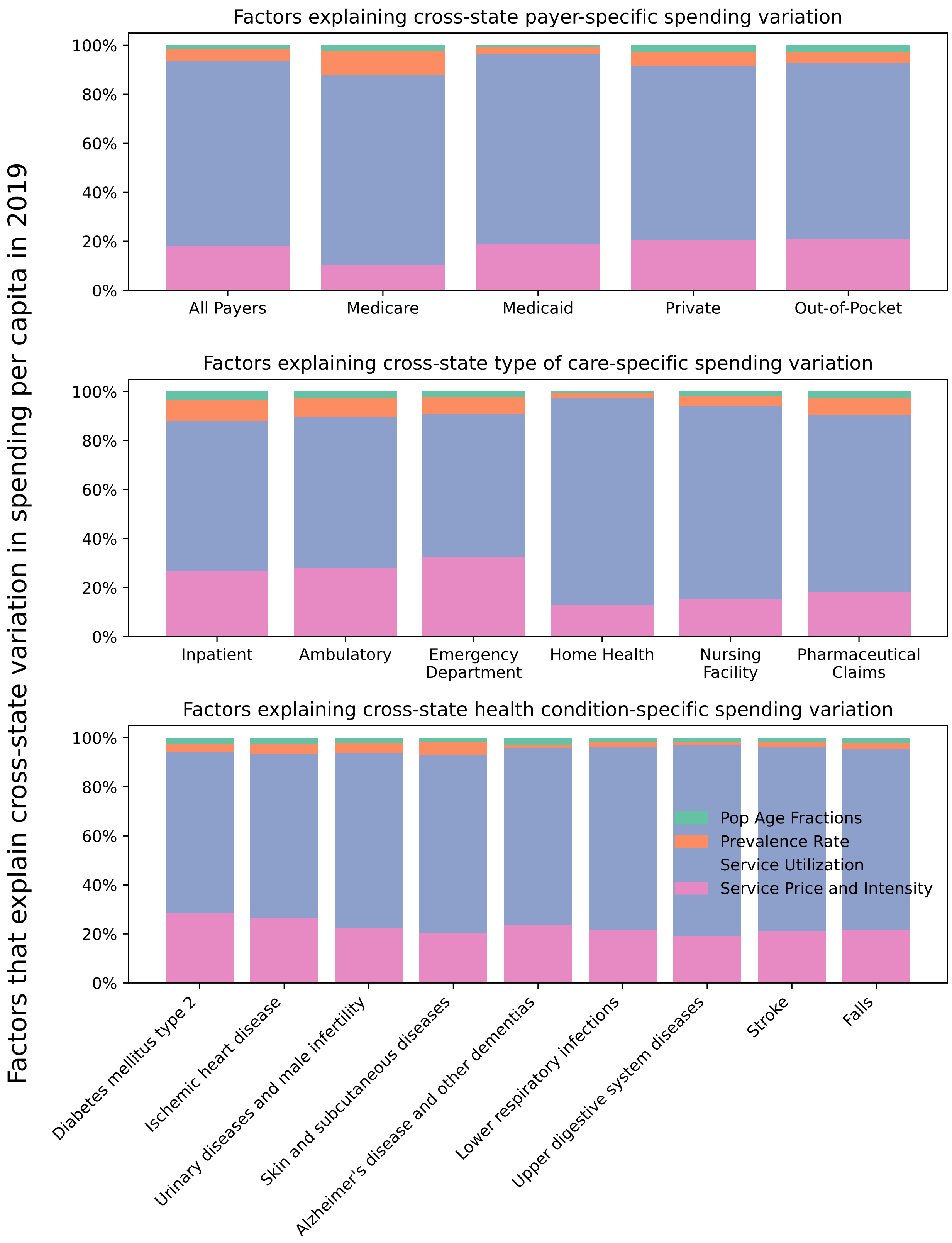

© 2025 Dieleman JL et al. JAMA Health Forum.

Further, several health conditions were excluded in our analysis due to restrictions in prevalence/incidence estimate availability. As such, we also ran a separate version of Shapley decomposition without prevalence/incidence using the full set of 148 health conditions originally included in the Disease Expenditure team study. This was accomplished by reducing the number of factors and modifying the definitions accordingly:

$$Age - Sex\ Population\ Proportion = \frac{Pop_{a,s,l}}{Pop_l}$$

$$Service\ Utilization = \frac{Encounters_{a,s,p,t,c,l}}{Pop_{a,s,l}}$$

$$Service\ Price\ and\ Intensity = \frac{Spending_{a,s,p,t,c,l}}{Encounters_{a,s,p,t,c,l}}$$

As a result, the overall linear model equation was also altered to become the following:

$$\ln\left(\frac{Spending_{a,s,\mathrm{p},\mathrm{t},c,l}}{Pop_l}\right) = \alpha_{a,s,p,t,c} + \beta_1 \ln\left(\frac{Pop_{a,s,l}}{Pop_l}\right) + \beta_2 \ln\left(\frac{Encounters_{a,s,p,t,c,l}}{Pop_{a,s,l}}\right) + \beta_3 \ln\left(\frac{Spending_{a,s,p,t,c,l}}{Encounters_{a,s,p,t,c,l}}\right) + \varepsilon_{a,s,p,t,c,l}$$

Again, observations were mean-centered by age-sex-payer-type of care-condition combination across all locations. The final dataset was regressed using all observations, rather than using all possible facets listed in Table 3.2.1. The Shapley decomposition of R-squared values for each factor is shown below in Figure 3.3.7.

© 2025 Dieleman JL et al. JAMA Health Forum.

Figure 3.3.7

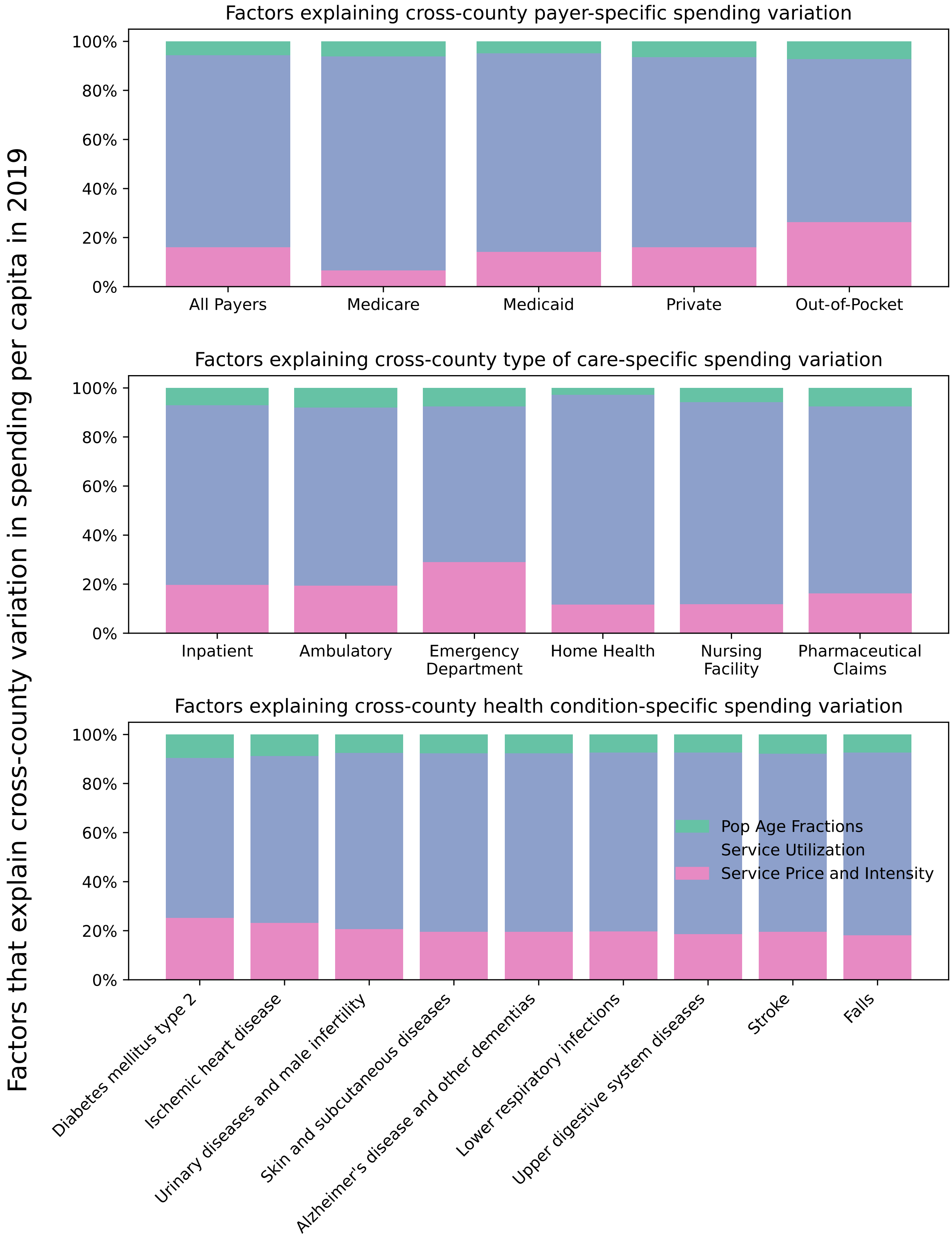

© 2025 Dieleman JL et al. JAMA Health Forum.

S4 Regression Analysis

S4.1 Overview

We calculated two metrics, service utilization and service price and intensity, and used bivariate linear regression to determine the influence of 8 covariates on each metric. These covariates were (1) rate of insured individuals, (2) median household income, (3) proportion of urban households, (4) number of individuals with medical doctorates (MDs) per capita, (5) rate of individuals with a bachelor's degree or above, (6) fraction of insured individuals that are have private insurance coverage, (7) fraction of MDs that serve as primary care providers (PCPs), and (8) fraction of individuals covered by Medicare that use Medicare Advantage. Each of these covariates was obtained as explained in Section 2.3 above.

The following bivariate linear regression equations were used in this analysis, representing an analysis of service utilization and service price and intensity, respectively:

$$\ln\left(\frac{Encounters_{a,s,p,t,c,l}}{Cases_{a,s,c,l}}\right) = \alpha_{a,s,p,t,c} + \beta_1 * X_l + \varepsilon_{a,s,p,t,c,l}$$

$$\ln\left(\frac{Spending_{a,s,p,t,c,l}}{Encounters_{a,s,p,t,c,l}}\right) = \alpha_{a,s,p,t,c} + \beta_1 * X_l + \varepsilon_{a,s,p,t,c,l}$$

Where X_l represents one of the location-specific covariates above. As before, individual regression models were created for each type of care, resulting in a total of 30 bivariate regression models for each of the equations above (6 types of care and 5 covariates).

The same definitions for service utilization as outlined in Section 3.1 were used for this regression analysis.

S4.2 Methodology

For the above regressions, *Cases* were taken from the USHD study and GBD 2021 and estimates of *Utilization* and *Spending* came from the Disease Expenditure 2019 study. Specific processing for the *Cases* variable is detailed in Section 2.2 above, which was undertaken prior to running regression models. Unlike previous analyses, these values were payer aggregated where possible. Individual regression models were run for each specific type of care, resulting in 6 models for each of the equations in section 4.1.

© 2025 Dieleman JL et al. JAMA Health Forum.

Covariate values were normalized to fall within the median and 75th percentile of each covariate range, to allow for a direct comparison of covariate values. This was calculated using the following equation:

$$Normalized\ Covariate\ Value = \frac{X - X_{med}}{X_{Q3} - X_{med}}$$

Where X represents the covariate value, X_{med} represents the median covariate value, and X_{Q3} represents the 75th percentile value. The normalized values were calculated at the location level and used in the final regression models.

We demeaned the dependent variable and each covariate by the age, sex, payer, type of care, and cause specific mean across counties, as in section 3.2.

The demeaned dependent variable was regressed on the normalized covariate values, and the corresponding coefficient estimates were recorded. Regressions were run individually for each type of care, resulting in type of care specific beta values. 50 draws of regressions were used in our analysis, applying Ruben's Rules to combine estimated data uncertainty and estimated parameter uncertainty. Additionally, to account for multiple hypothesis testing, betas were adjusted using the Bonferroni correction with the number of tests being 60 (six types of care, five covariates, two metrics).

S4.3 Additional results

The exact estimated values for the covariates shown in Figure 3, including lower and upper confidence intervals, are available in Table 4.3.1 below.

Table 4.3.1

Metric	Type of Care	Covariate	Estimated Value	Lower CI	Upper CI
Service Utilization	Ambulatory	Insured rate	0.0776	0.0356	0.1196
Service Utilization	Ambulatory	Median household income	0.0435	0.0213	0.0658
Service Utilization	Ambulatory	Proportion urban households	0.0200	-0.0494	0.0895
Service Utilization	Ambulatory	MDs per capita	0.0082	-0.0090	0.0254
Service Utilization	Ambulatory	Bachelor's degree+ rate	0.0384	0.0090	0.0677
Service Utilization	Ambulatory	Fraction of insured that are privately insured	0.0200	-0.0013	0.0413

© 2025 Dieleman JL et al. JAMA Health Forum.

Service Utilization	Ambulatory	Fraction of MDs that are Primary Care Providers	-0.0178	-0.0738	0.0381
Service Utilization	Ambulatory	Fraction of Medicare that is Medicare Advantage	-0.0187	-0.0315	-0.0059
Service Utilization	Emergency Department	Insured rate	0.0495	-0.0135	0.1126
Service Utilization	Emergency Department	Median household income	-0.0092	-0.0446	0.0261
Service Utilization	Emergency Department	Proportion urban households	-0.0456	-0.1072	0.0159
Service Utilization	Emergency Department	MDs per capita	0.0062	-0.0131	0.0255
Service Utilization	Emergency Department	Bachelor's degree+ rate	-0.0202	-0.0593	0.0190
Service Utilization	Emergency Department	Fraction of insured that are privately insured	-0.0776	-0.1175	-0.0376
Service Utilization	Emergency Department	Fraction of MDs that are Primary Care Providers	0.0050	-0.0466	0.0565
Service Utilization	Emergency Department	Fraction of Medicare that is Medicare Advantage	-0.0161	-0.0324	0.0003
Service Utilization	Home Health	Insured rate	0.1040	0.0538	0.1541
Service Utilization	Home Health	Median household income	0.0713	0.0507	0.0920
Service Utilization	Home Health	Proportion urban households	0.1218	0.0548	0.1888
Service Utilization	Home Health	MDs per capita	0.0394	0.0234	0.0554
Service Utilization	Home Health	Bachelor's degree+ rate	0.0691	0.0418	0.0963
Service Utilization	Home Health	Fraction of insured that are privately insured	-0.0670	-0.0989	-0.0352
Service Utilization	Home Health	Fraction of MDs that are Primary Care Providers	-0.1609	-0.2384	-0.0835
Service Utilization	Home Health	Fraction of Medicare that is	0.0039	-0.0383	0.0461

© 2025 Dieleman JL et al. JAMA Health Forum.

		Medicare Advantage			
Service Utilization	Inpatient	Insured rate	0.0560	-0.0002	0.1122
Service Utilization	Inpatient	Median household income	0.0127	-0.0352	0.0606
Service Utilization	Inpatient	Proportion urban households	-0.0684	-0.1673	0.0306
Service Utilization	Inpatient	MDs per capita	-0.0124	-0.0316	0.0069
Service Utilization	Inpatient	Bachelor's degree+ rate	-0.0134	-0.0718	0.0450
Service Utilization	Inpatient	Fraction of insured that are privately insured	-0.0036	-0.0590	0.0519
Service Utilization	Inpatient	Fraction of MDs that are Primary Care Providers	0.0626	-0.0124	0.1376
Service Utilization	Inpatient	Fraction of Medicare that is Medicare Advantage	-0.0487	-0.0717	-0.0257
Service Utilization	Nursing Facility	Insured rate	0.1612	0.1160	0.2064
Service Utilization	Nursing Facility	Median household income	0.0622	0.0393	0.0852
Service Utilization	Nursing Facility	Proportion urban households	0.0609	0.0073	0.1146
Service Utilization	Nursing Facility	MDs per capita	0.0289	0.0124	0.0455
Service Utilization	Nursing Facility	Bachelor's degree+ rate	0.0792	0.0504	0.1081
Service Utilization	Nursing Facility	Fraction of insured that are privately insured	0.0155	-0.0106	0.0416
Service Utilization	Nursing Facility	Fraction of MDs that are Primary Care Providers	-0.0401	-0.0877	0.0075
Service Utilization	Nursing Facility	Fraction of Medicare that is Medicare Advantage	-0.0442	-0.0772	-0.0113
Service Utilization	Pharmaceutical Claims	Insured rate	0.0264	-0.0021	0.0549

© 2025 Dieleman JL et al. JAMA Health Forum.

Service Utilization	Pharmaceutical Claims	Median household income	0.0373	0.0122	0.0623
Service Utilization	Pharmaceutical Claims	Proportion urban households	0.0016	-0.0363	0.0395
Service Utilization	Pharmaceutical Claims	MDs per capita	0.0024	-0.0093	0.0142
Service Utilization	Pharmaceutical Claims	Bachelor's degree+ rate	0.0279	0.0052	0.0506
Service Utilization	Pharmaceutical Claims	Fraction of insured that are privately insured	0.0470	0.0137	0.0802
Service Utilization	Pharmaceutical Claims	Fraction of MDs that are Primary Care Providers	-0.0135	-0.0513	0.0244
Service Utilization	Pharmaceutical Claims	Fraction of Medicare that is Medicare Advantage	-0.0271	-0.0473	-0.0069
Service Price and Intensity	Ambulatory	Insured rate	-0.0012	-0.0149	0.0125
Service Price and Intensity	Ambulatory	Median household income	0.0274	0.0170	0.0378
Service Price and Intensity	Ambulatory	Proportion urban households	0.0565	0.0359	0.0771
Service Price and Intensity	Ambulatory	MDs per capita	0.0086	0.0038	0.0135
Service Price and Intensity	Ambulatory	Bachelor's degree+ rate	0.0363	0.0213	0.0514
Service Price and Intensity	Ambulatory	Fraction of insured that are privately insured	0.0434	0.0206	0.0662
Service Price and Intensity	Ambulatory	Fraction of MDs that are Primary Care Providers	-0.0370	-0.0554	-0.0185
Service Price and Intensity	Ambulatory	Fraction of Medicare that is Medicare Advantage	0.0131	0.0079	0.0183
Service Price and Intensity	Emergency Department	Insured rate	-0.0009	-0.0394	0.0377
Service Price and Intensity	Emergency Department	Median household income	0.0618	0.0341	0.0896

© 2025 Dieleman JL et al. JAMA Health Forum.

Service Price and Intensity	Emergency Department	Proportion urban households	0.0180	-0.0332	0.0693
Service Price and Intensity	Emergency Department	MDs per capita	-0.0100	-0.0241	0.0042
Service Price and Intensity	Emergency Department	Bachelor's degree+ rate	0.0604	0.0246	0.0963
Service Price and Intensity	Emergency Department	Fraction of insured that are privately insured	0.1430	0.0786	0.2074
Service Price and Intensity	Emergency Department	Fraction of MDs that are Primary Care Providers	0.0264	-0.0168	0.0697
Service Price and Intensity	Emergency Department	Fraction of Medicare that is Medicare Advantage	-0.0191	-0.0331	-0.0052
Service Price and Intensity	Home Health	Insured rate	-0.0078	-0.0307	0.0151
Service Price and Intensity	Home Health	Median household income	-0.0084	-0.0192	0.0024
Service Price and Intensity	Home Health	Proportion urban households	0.0024	-0.0227	0.0275
Service Price and Intensity	Home Health	MDs per capita	-0.0051	-0.0129	0.0027
Service Price and Intensity	Home Health	Bachelor's degree+ rate	-0.0085	-0.0226	0.0055
Service Price and Intensity	Home Health	Fraction of insured that are privately insured	-0.0003	-0.0158	0.0152
Service Price and Intensity	Home Health	Fraction of MDs that are Primary Care Providers	0.0196	-0.0051	0.0443
Service Price and Intensity	Home Health	Fraction of Medicare that is Medicare Advantage	0.0138	0.0028	0.0248
Service Price and Intensity	Inpatient	Insured rate	0.0284	0.0123	0.0446
Service Price and Intensity	Inpatient	Median household income	0.0429	0.0238	0.0620
Service Price and Intensity	Inpatient	Proportion urban households	0.0972	0.0564	0.1380

© 2025 Dieleman JL et al. JAMA Health Forum.

Service Price and Intensity	Inpatient	MDs per capita	0.0194	0.0095	0.0294
Service Price and Intensity	Inpatient	Bachelor's degree+ rate	0.0541	0.0265	0.0816
Service Price and Intensity	Inpatient	Fraction of insured that are privately insured	0.0256	0.0083	0.0429
Service Price and Intensity	Inpatient	Fraction of MDs that are Primary Care Providers	-0.0830	-0.1162	-0.0498
Service Price and Intensity	Inpatient	Fraction of Medicare that is Medicare Advantage	0.0154	0.0075	0.0232
Service Price and Intensity	Nursing Facility	Insured rate	-0.0361	-0.0573	-0.0149
Service Price and Intensity	Nursing Facility	Median household income	-0.0016	-0.0130	0.0098
Service Price and Intensity	Nursing Facility	Proportion urban households	0.0062	-0.0147	0.0271
Service Price and Intensity	Nursing Facility	MDs per capita	-0.0007	-0.0078	0.0065
Service Price and Intensity	Nursing Facility	Bachelor's degree+ rate	-0.0068	-0.0185	0.0050
Service Price and Intensity	Nursing Facility	Fraction of insured that are privately insured	-0.0064	-0.0298	0.0170
Service Price and Intensity	Nursing Facility	Fraction of MDs that are Primary Care Providers	-0.0230	-0.0445	-0.0015
Service Price and Intensity	Nursing Facility	Fraction of Medicare that is Medicare Advantage	0.0175	0.0046	0.0304
Service Price and Intensity	Pharmaceutical Claims	Insured rate	0.0026	-0.0079	0.0131
Service Price and Intensity	Pharmaceutical Claims	Median household income	0.0177	0.0090	0.0263
Service Price and Intensity	Pharmaceutical Claims	Proportion urban households	0.0410	0.0221	0.0600
Service Price and Intensity	Pharmaceutical Claims	MDs per capita	0.0070	0.0017	0.0123

© 2025 Dieleman JL et al. JAMA Health Forum.

Service Price and Intensity	Pharmaceutical Claims	Bachelor's degree+ rate	0.0236	0.0119	0.0353
Service Price and Intensity	Pharmaceutical Claims	Fraction of insured that are privately insured	0.0231	0.0064	0.0399
Service Price and Intensity	Pharmaceutical Claims	Fraction of MDs that are Primary Care Providers	-0.0315	-0.0478	-0.0151
Service Price and Intensity	Pharmaceutical Claims	Fraction of Medicare that is Medicare Advantage	0.0166	0.0076	0.0255

© 2025 Dieleman JL et al. JAMA Health Forum.

S5 Das Gupta Decomposition

S5.1 Overview

We calculated spending per capita at state and national levels, decomposing the state level spending rate on the national spending rate in 2019 by four key factors associated with health care spending using methods previously described in Dieleman et al. (2017). These factors were (1) proportion of the total population in a particular age-sex group, (2) health condition incidence (for all injuries and cancers) or prevalence (for all other diseases), (3) service utilization, and (4) service price and intensity, including the use of new technologies. For each age-sex-payer-type of care-condition-location combination we calculated each of the four factors as:

$$Age - Sex\ Population\ Proportion = \frac{Pop_{a,s,l}}{Pop_l}$$
$$Condition\ Incidence\ or\ Prevalence\ = \frac{Cases_{a,s,c,l}}{Pop_{a,s,l}}$$
$$Service\ Utilization = \frac{Encounters_{a,s,p,t,c,l}}{Cases_{a,s,c,l}}$$
$$Service\ Price\ and\ Intensity = \frac{Spending_{a,s,p,t,c,l}}{Encounters_{a,s,p,t,c,l}}$$

Spending per capita was then expressed as a rate of all four factors, as shown below.

$$\frac{Spending_{a,s,c,l}}{Pop_l} = \frac{Pop_{a,s,l}}{Pop_l} \times \frac{Cases_{a,s,c,l}}{Pop_{a,s,l}} \times \frac{Encounters_{a,s,p,t,c,l}}{Cases_{a,s,c,l}} \times \frac{Spending_{a,s,p,t,c,l}}{Encounters_{a,s,p,t,c,l}}$$

The same definitions for service utilization as outlined in Section 3.1 were used for Das Gupta decomposition.

To estimate the relative effect of each of the four factors on the difference in spending per capita between the state and national level, we conducted a decomposition analysis using the methods described by Das Gupta (1993).

S5.2 Methodology

Estimates of the *Pop* variable above were obtained from the USHD study, *Cases* were taken from the USHD study and GBD 2021 and estimates of *Utilization* and *Spending* came from the Disease Expenditure 2019 study. Specific processing for the *Cases* variable is detailed in Section 2.2 above, which was undertaken prior to Das Gupta decomposition.

© 2025 Dieleman JL et al. JAMA Health Forum.

The four factors were then calculated using the equations noted above for each combination of age-sex-payer-type of care-condition for all states and at the national level in 2019. State and type of care specific factor values were then decomposed against corresponding national and type of care specific factors values. That is, each age-sex-payer-type of care-condition-location specific factor value was decomposed against the corresponding age-sex-payer-type of care-condition factor value at the national level.

Decomposition followed the standard four factor method detailed in Das Gupta (1993). The resulting effect of each factor from decomposition was then summed to the type of care-location level. These aggregated effect values were then divided by the type of care specific national rate to compare state spending per capita relative to the national spending per capita. Results were averaged across 50 draws of data.

S5.3 Additional results

To analyze the stability of our Das Gupta decomposition results across time, we repeated the methods above for two additional years of data, 2010 and 2015. These results are shown below in Figure 5.3.1 and Figure 5.3.2, respectively. A set of full results for all states in 2019 is also shown in Figure 5.3.3.

© 2025 Dieleman JL et al. JAMA Health Forum.

Figure 5.3.1

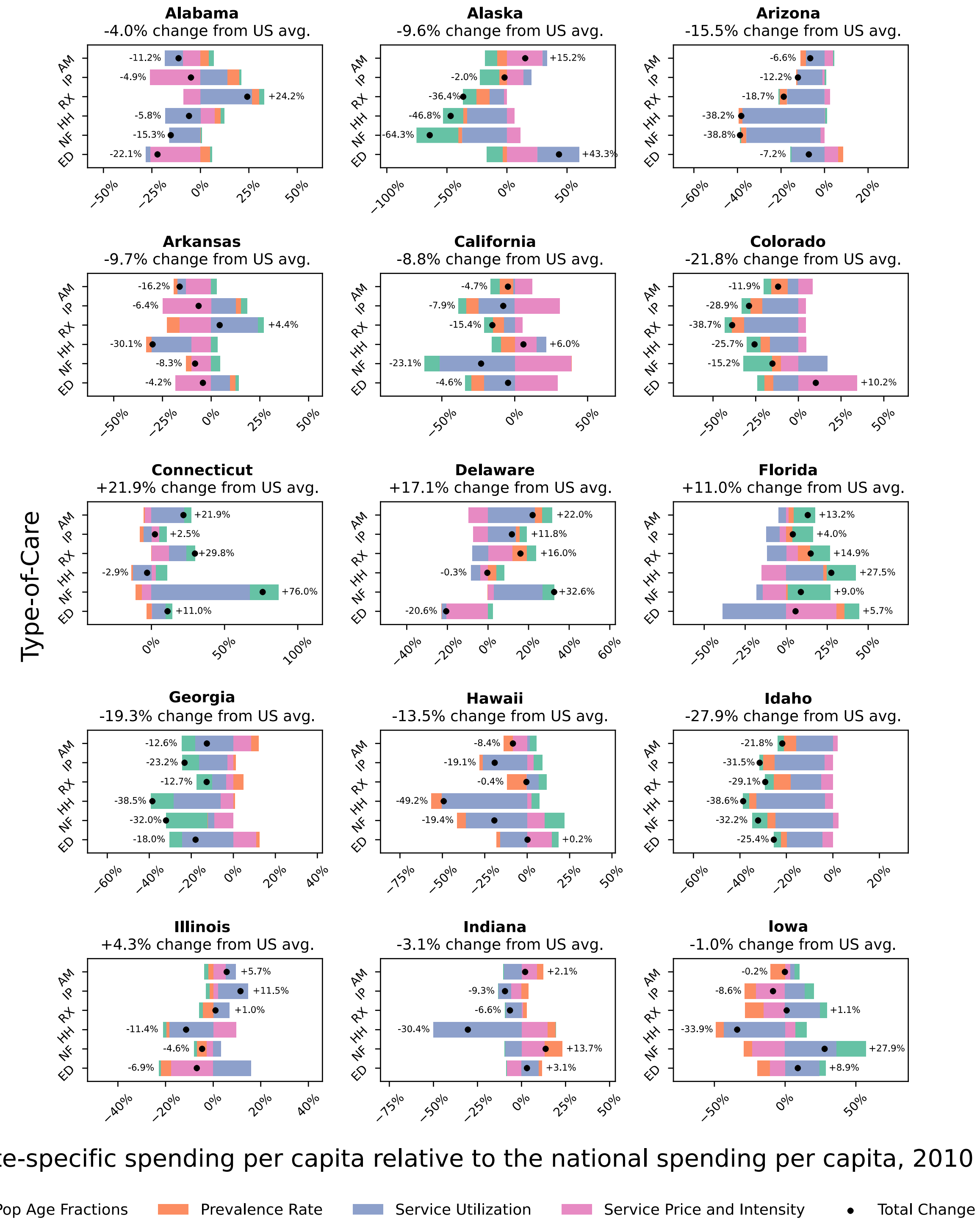

© 2025 Dieleman JL et al. JAMA Health Forum.

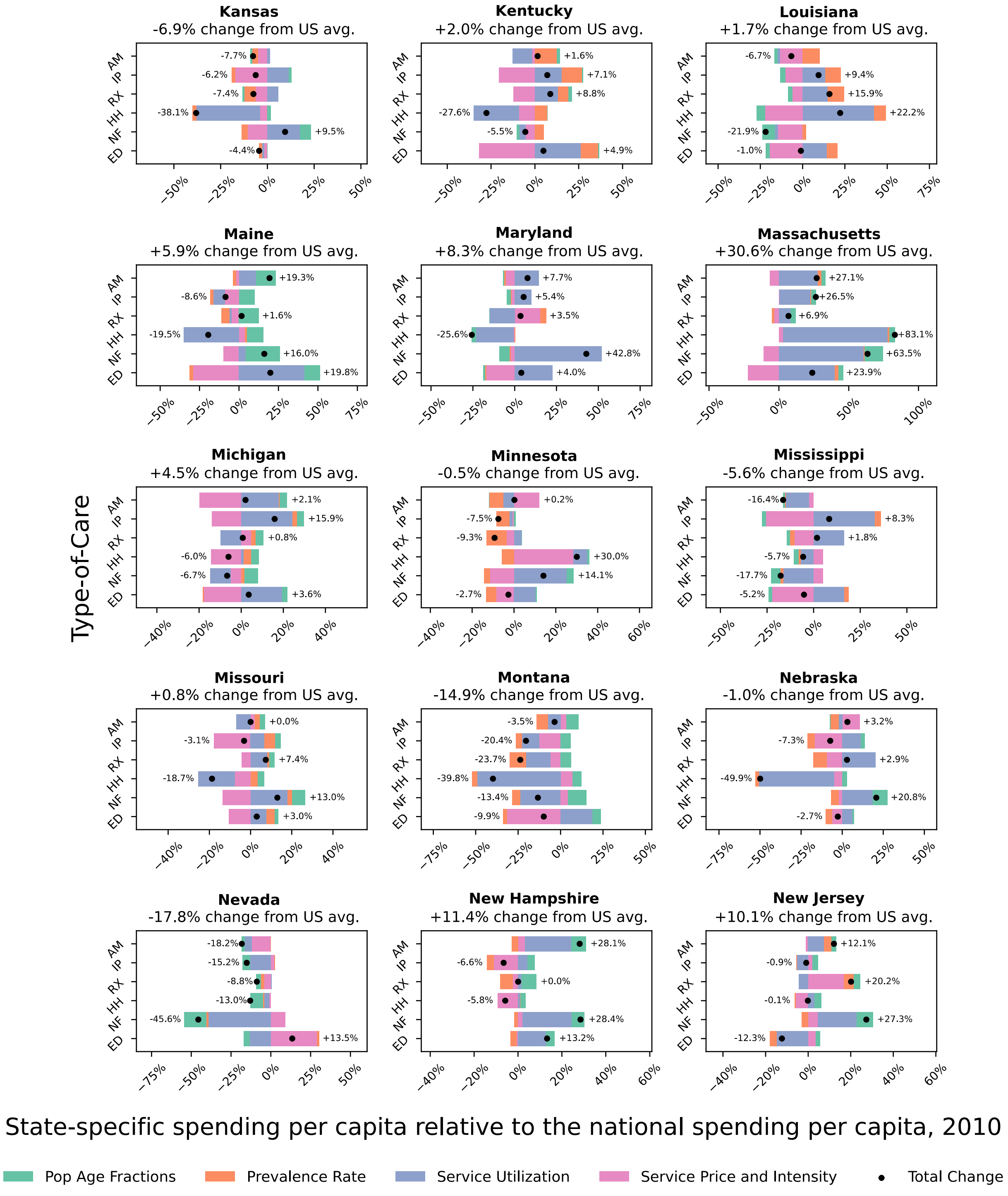

© 2025 Dieleman JL et al. JAMA Health Forum.

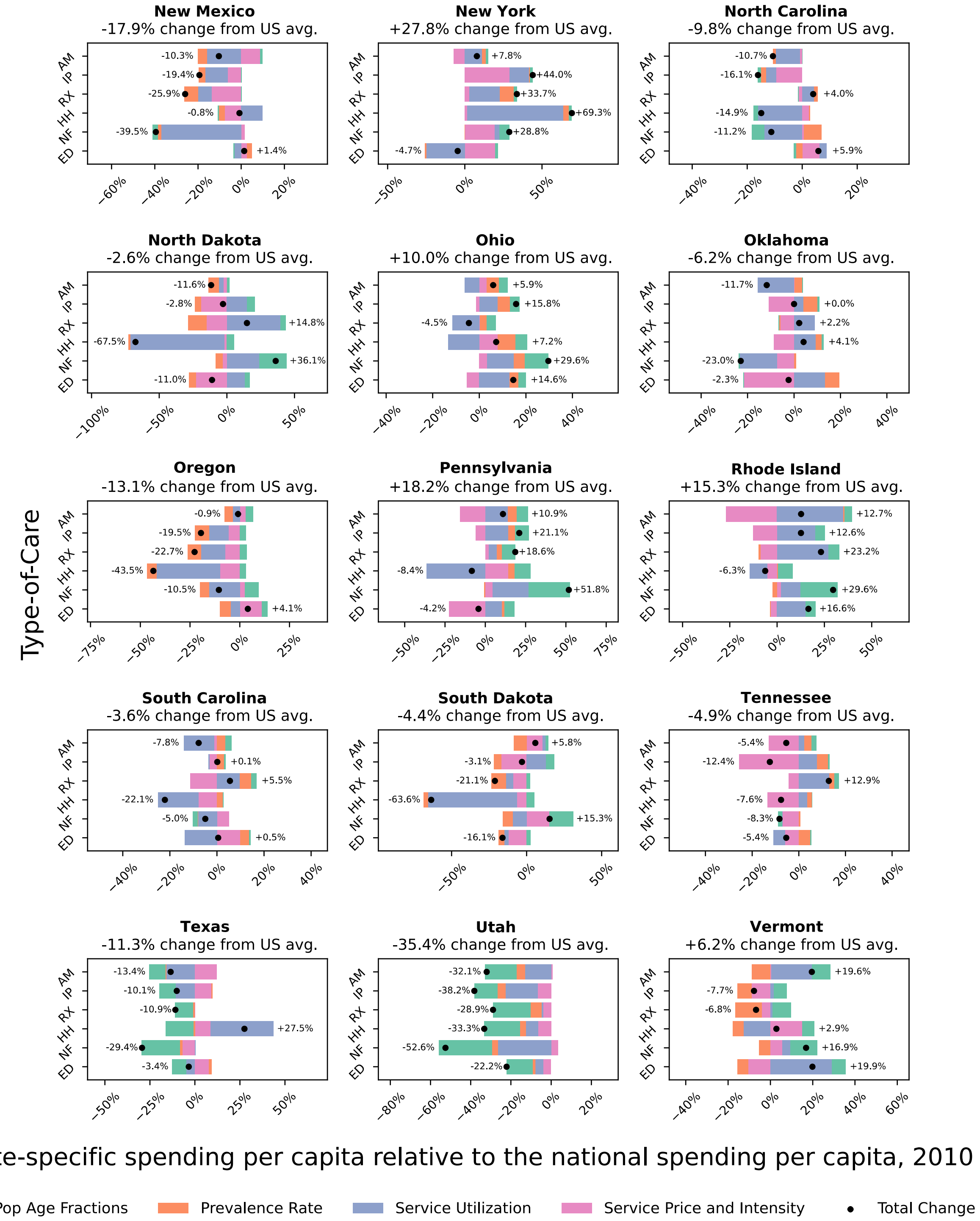

© 2025 Dieleman JL et al. JAMA Health Forum.

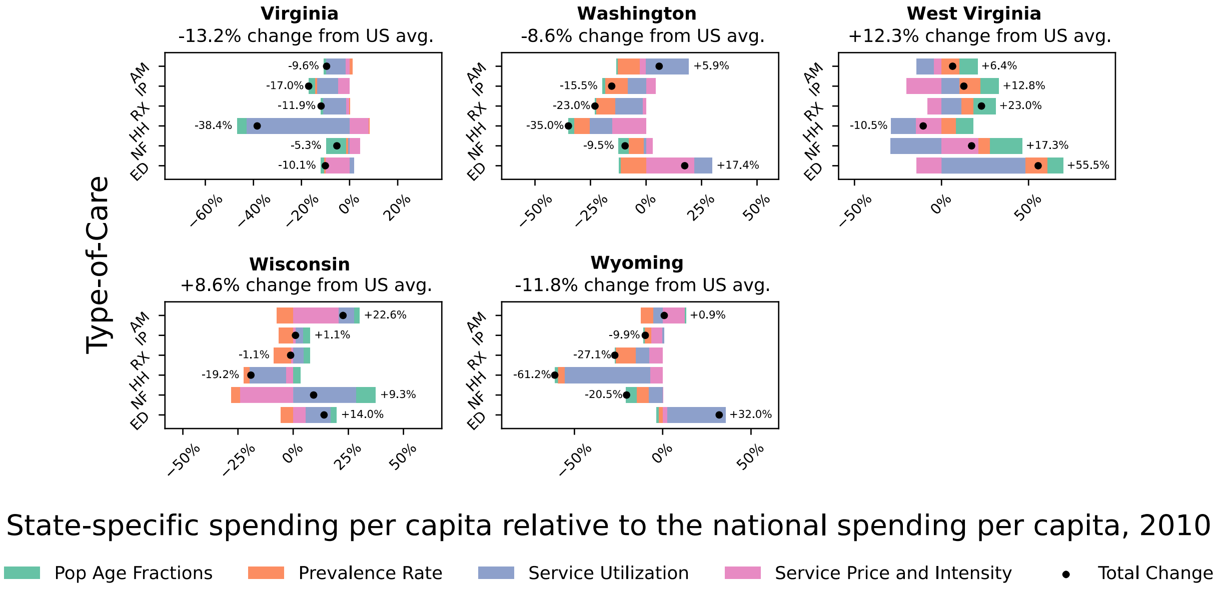

© 2025 Dieleman JL et al. JAMA Health Forum.

Figure 5.3.2

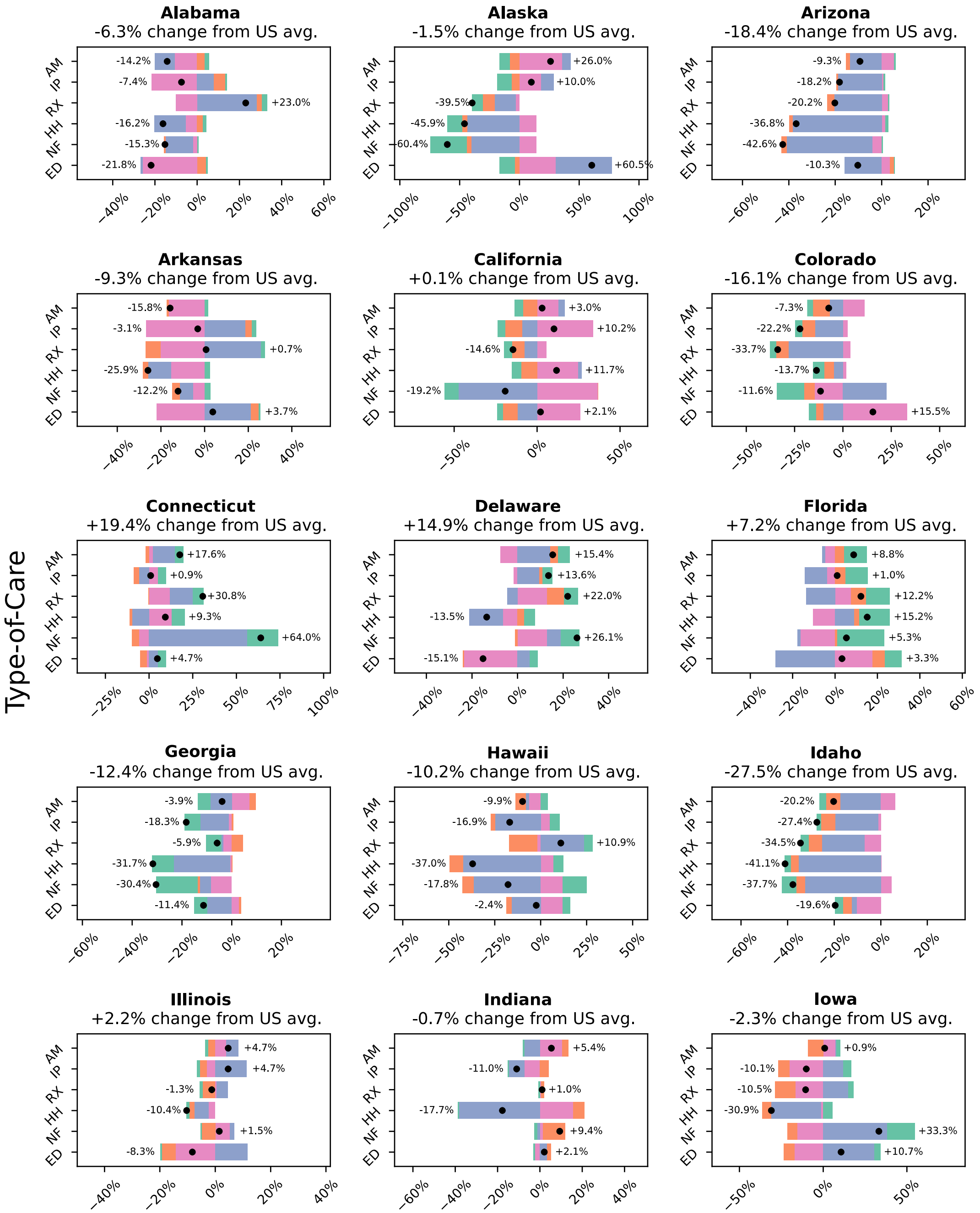

State-specific spending per capita relative to the national spending per capita, 2015

Pop Age Fractions   Prevalence Rate   Service Utilization   Service Price and Intensity   • Total Change

© 2025 Dieleman JL et al. JAMA Health Forum.

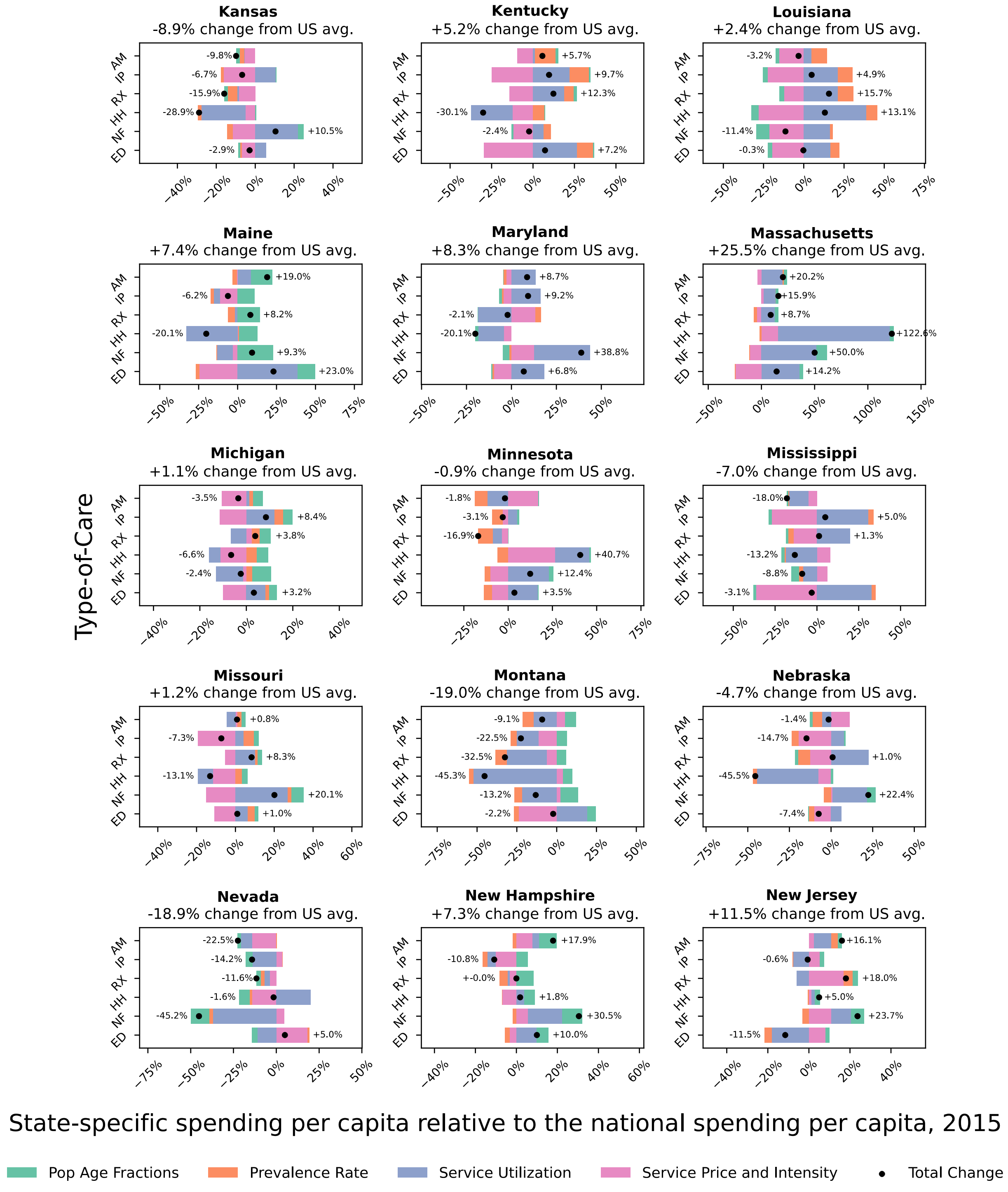

© 2025 Dieleman JL et al. JAMA Health Forum.

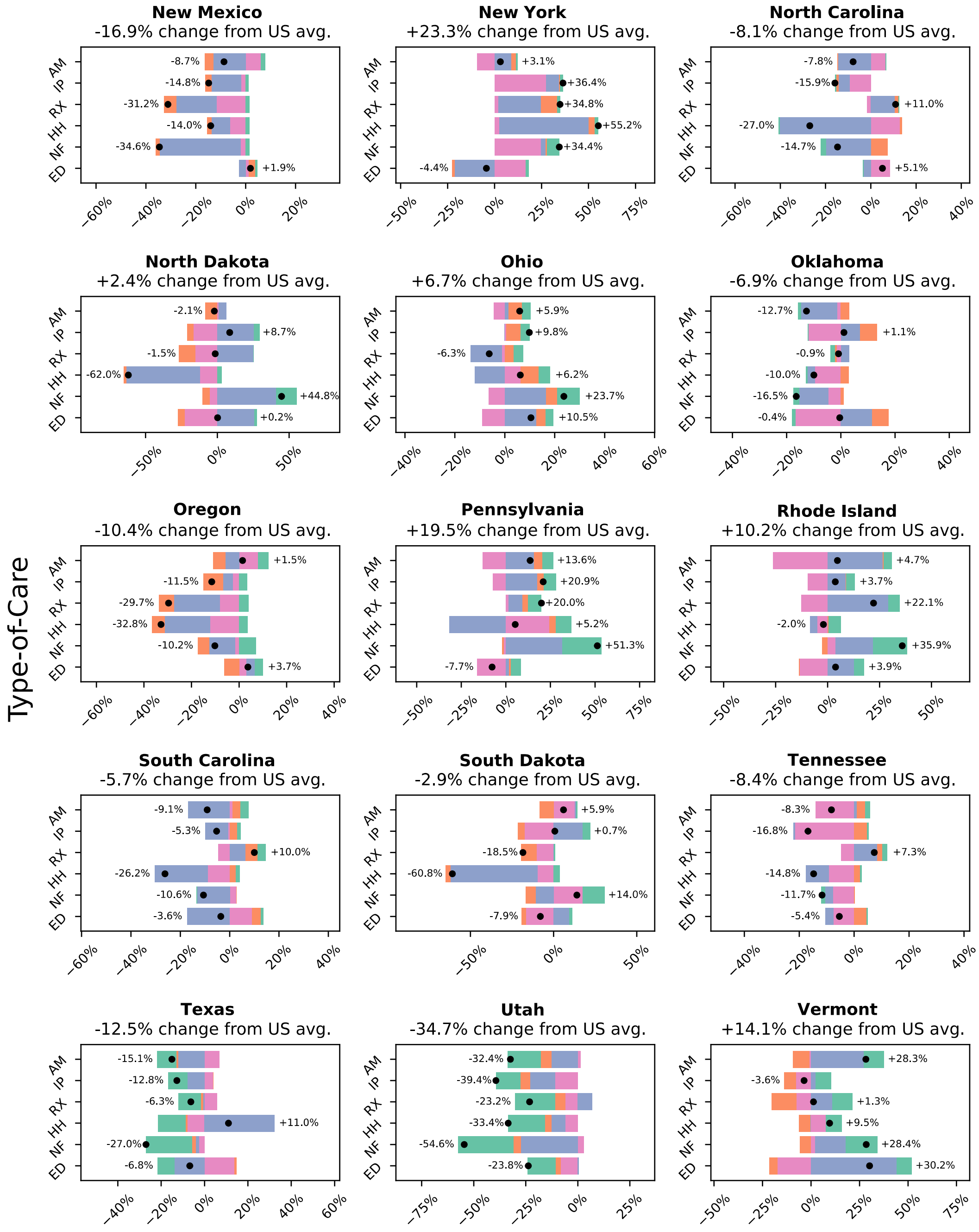

State-specific spending per capita relative to the national spending per capita, 2015

Pop Age Fractions · Prevalence Rate · Service Utilization · Service Price and Intensity · Total Change

© 2025 Dieleman JL et al. JAMA Health Forum.

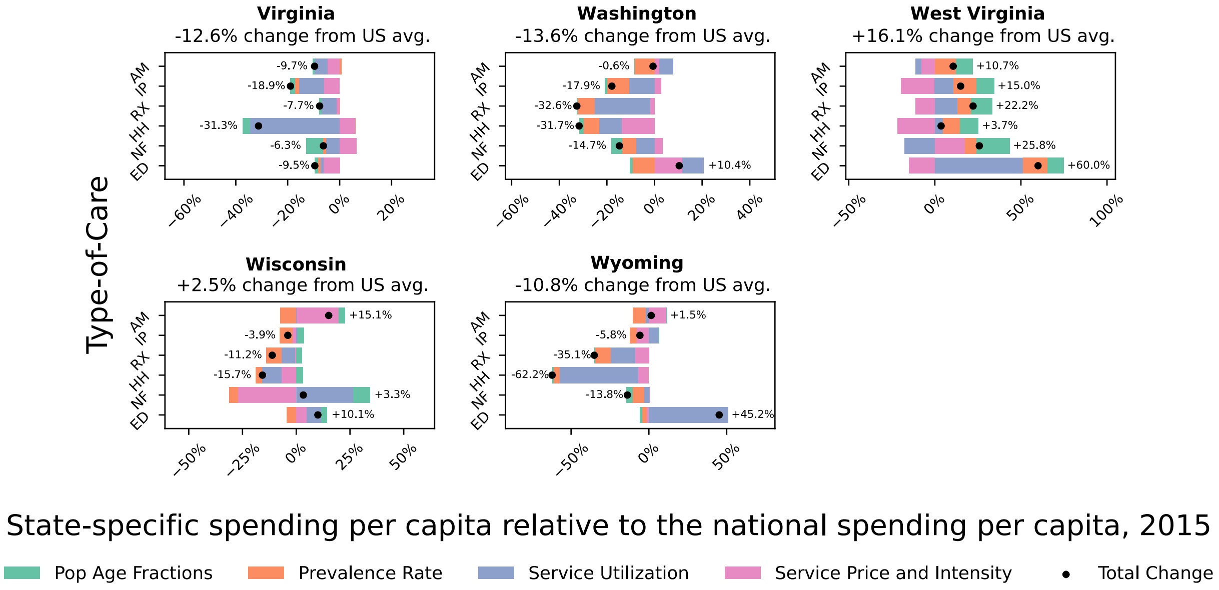

© 2025 Dieleman JL et al. JAMA Health Forum.

Figure 5.3.3

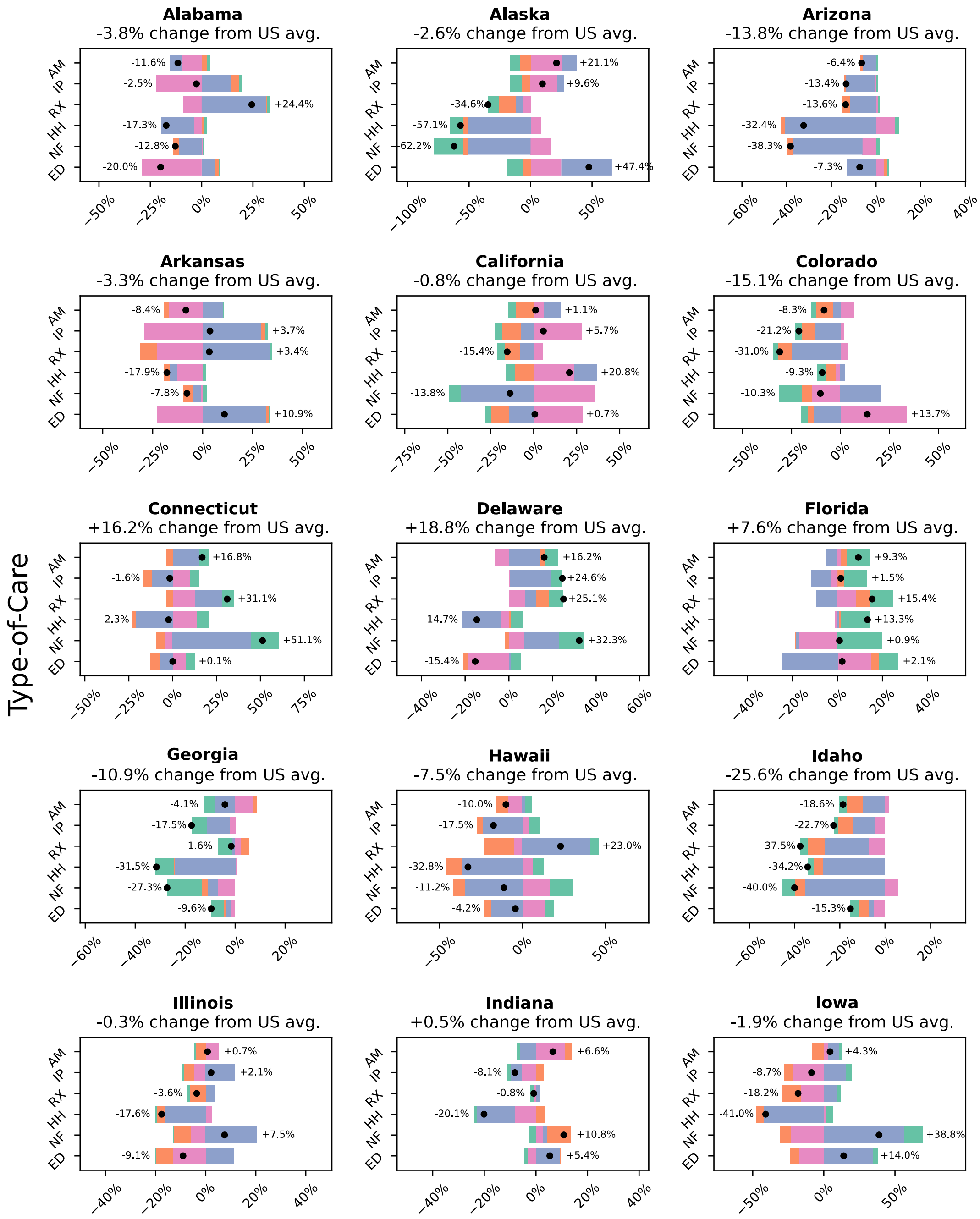

State-specific spending per capita relative to the national spending per capita, 2019

Pop Age Fractions | Prevalence Rate | Service Utilization | Service Price and Intensity | • Total Change

© 2025 Dieleman JL et al. JAMA Health Forum.

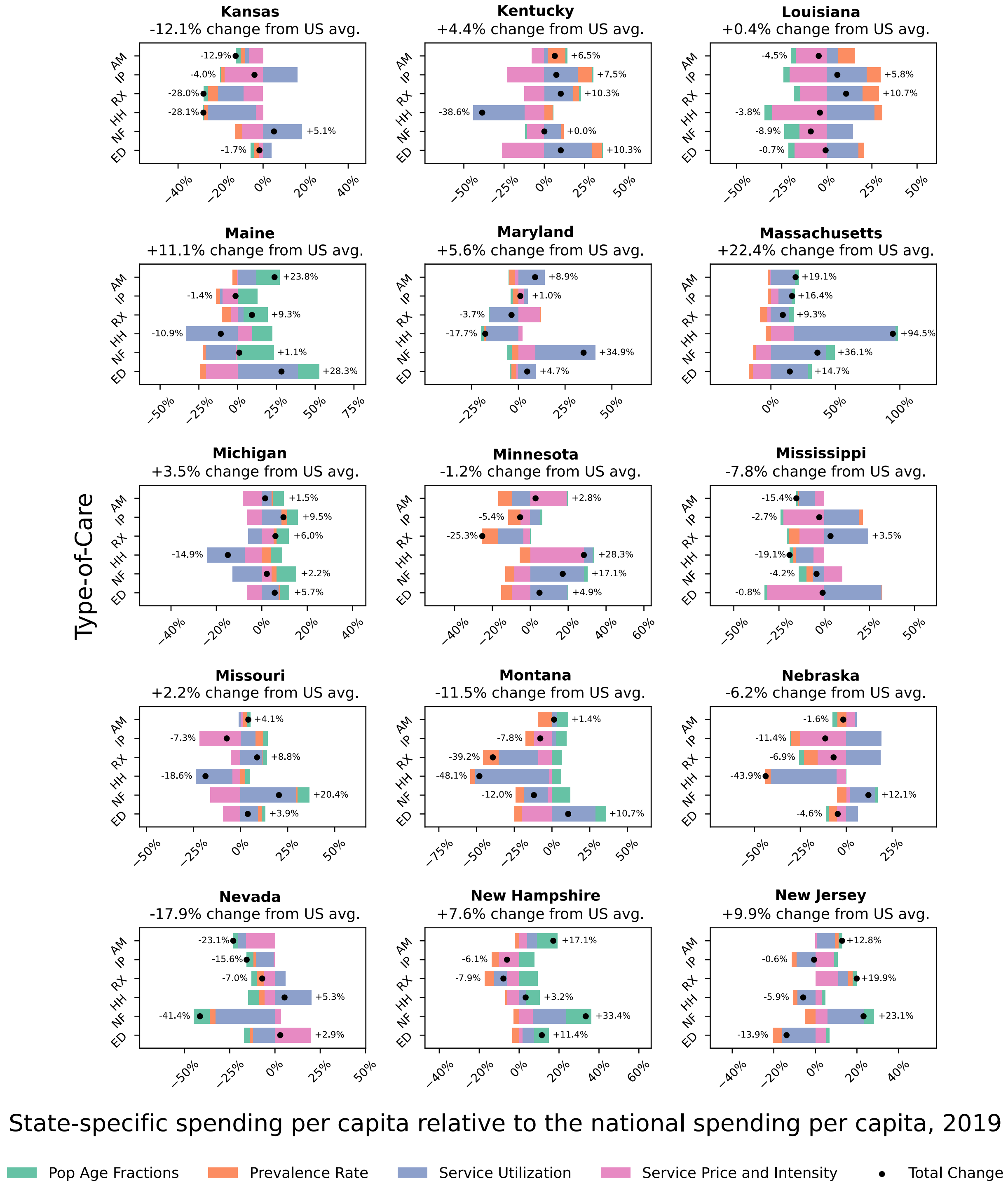

© 2025 Dieleman JL et al. JAMA Health Forum.

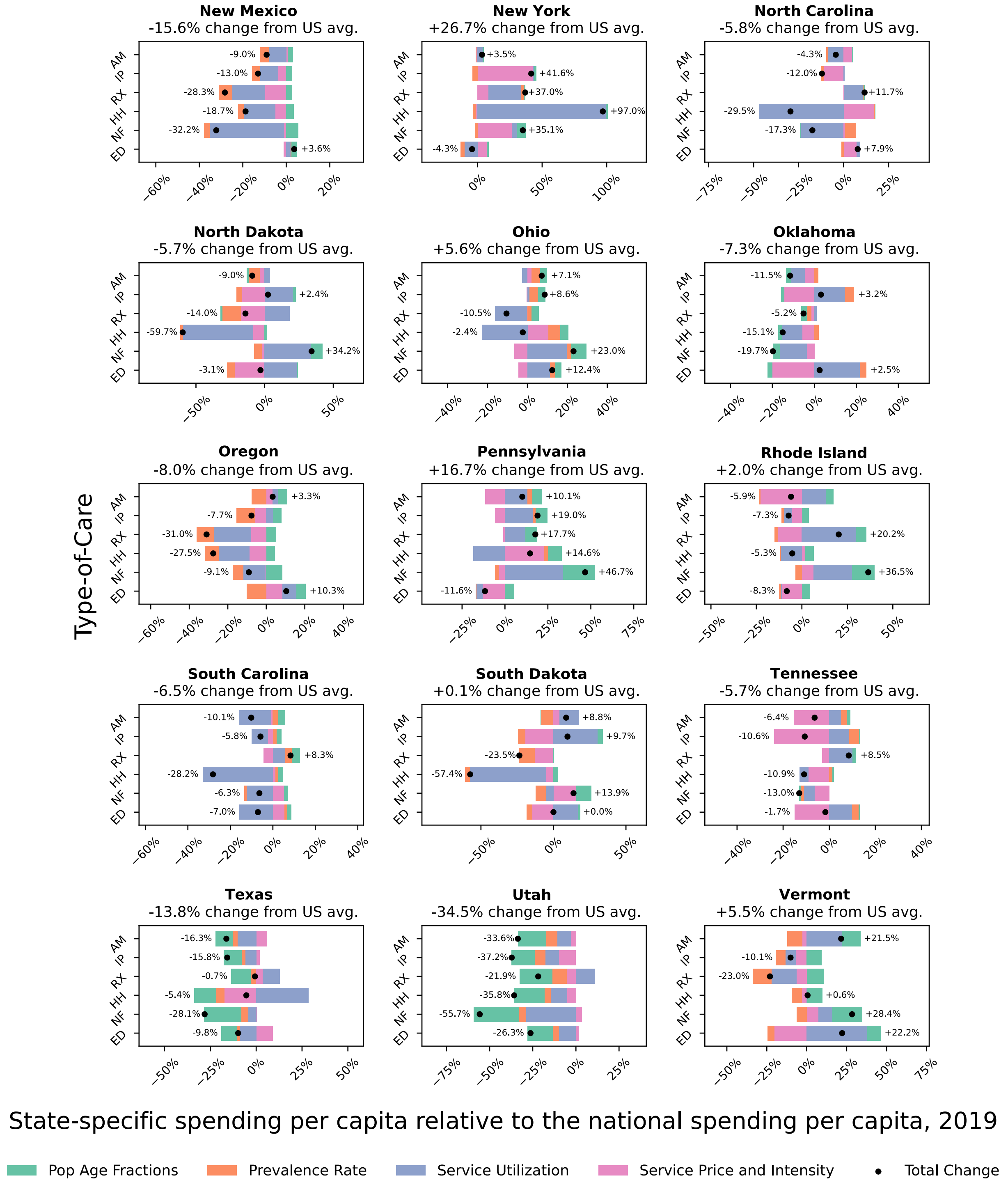

© 2025 Dieleman JL et al. JAMA Health Forum.

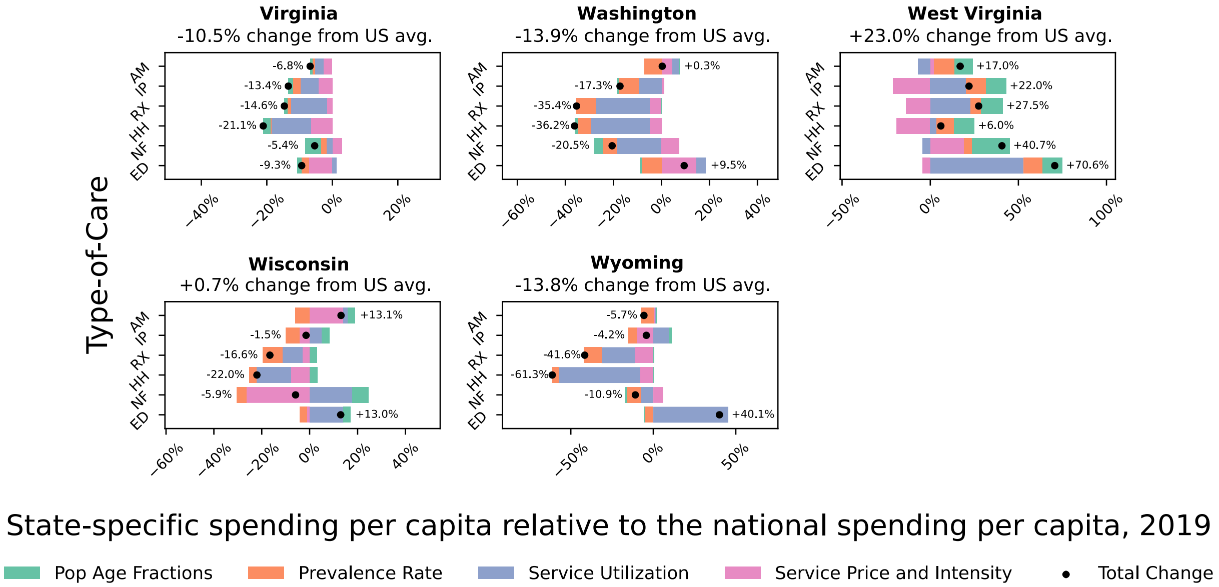

© 2025 Dieleman JL et al. JAMA Health Forum.

S6 GATHER Compliance

This study complies with the Guidelines for Accurate and Transparent Health Estimates Reporting (GATHER) recommendations. We have documented the steps involved in our analytical procedures and detailed the data sources used. See Table 6.1 for the GATHER checklist. The GATHER recommendations can be found here: http://gather-statement.org/

Table 6.1

#	GATHER checklist item	Description of compliance	Reference
Objectives and funding			
1	Define the indicators, populations, and time periods for which estimates were made.	Narrative provided in paper and methods appendix describing<br><br>indicators, definitions, and populations	Main text (Methods) and methods appendix
2	List the funding sources for the work.	Funding sources listed in paper	Main text (Acknowledgments)
Data Inputs			
For all data inputs from multiple sources that are synthesized as part of the study:			
3	Describe how the data were identified and how the data were accessed.	Narrative provided in paper and methods appendix describing data- seeking methods	Main text (Methods) and methods appendix
4	Specify the inclusion and exclusion criteria. Identify all ad-hoc exclusions.	Narrative provided in paper and methods appendix describing inclusion and exclusion criteria	Main text (Methods) and methods appendix

© 2025 Dieleman JL et al. JAMA Health Forum.

5	Provide information on all included data sources and their main characteristics. For each data source used, report reference information or contact name/institution, population represented, data collection method, year(s) of data collection, sex and age range, diagnostic criteria or measurement method, and sample<br><br>size, as relevant.	Metadata for data sources by component, activity, geography, currency, currency year, and income classification will be available through an interactive, online data record	Link to the GHDx to be provided upon publication.

6	Identify and describe any categories of input data that have potentially important biases (e.g., based on characteristics listed in item 5).	Summary of known biases included in paper narrative	Main text (Limitations)
For data inputs that contribute to the analysis but were not synthesized as part of the study:			
7	Describe and give sources for any other data inputs.	Will be included in GHDx link	Link to the GHDx to be provided upon publication.
For all data inputs:			

© 2025 Dieleman JL et al. JAMA Health Forum.

8	Provide all data inputs in a file format from which data can be efficiently extracted (e.g., a spreadsheet as opposed to a PDF), including all relevant meta-data listed in item 5. For any data inputs that cannot be shared due to ethical or legal reasons, such as third-party ownership, provide a contact name or the name of the institution that retains the right<br><br>to the data.	Downloads of input data available through online tools such as the Global Health Data Exchange website	Online data<br><br>visualization tools and the Global Health Data Exchange, http://ghdx.healthdata. org
Data analysis			
9	Provide a conceptual overview of the data analysis method. A diagram may be helpful.	Write ups of the overall methodological processes, as well as cause-specific modeling processes, have been provided	Main text (Methods) and methods appendix
10	Provide a detailed description of all steps of the analysis, including mathematical formulae. This description should cover, as relevant, data cleaning, data pre-processing, data adjustments and weighting of data sources, and mathematical or	Corresponding methodological write-ups have been provided	Main text (Methods) and methods appendix

© 2025 Dieleman JL et al. JAMA Health Forum.

	statistical model(s).		
11	Describe how candidate models were<br><br>evaluated and how the final model(s) were selected.	Details on evaluation of model performance have been provided	Methods appendix
12	Provide the results of an evaluation of model performance, if done, as well as the results of any relevant<br><br>sensitivity analysis.	Details on evaluation of model performance have been provided	Methods appendix
13	Describe methods for calculating uncertainty of the estimates. State which sources of uncertainty were, and were not, accounted for in the uncertainty analysis.	Details on uncertainty calculations have been provided	Methods appendix
14	State how analytic or statistical source code used to generate estimates can be accessed.	Access statement provided	Code is provided in an online repository, code will be provided upon publication.
Results and Discussion			

© 2025 Dieleman JL et al. JAMA Health Forum.

15	Provide published estimates in a file format from which data can be efficiently extracted.	Results are available through the<br><br>Global Health Data Exchange	Link to the GHDx to be provided upon publication.
16	Report a quantitative measure of the uncertainty of the estimates (e.g. uncertainty intervals).	Uncertainty intervals are provided with all results	Main text, methods appendix, and online data tools (the Global Health Data Exchange, link to the GHDx to be provided upon publication.)
17	Interpret results in light of existing evidence. If updating a previous set of estimates, describe the reasons for changes in estimates.	Discussion of methodological differences between our estimates and other available evidence provided in the paper and methods appendix	Main text (Methods and Discussion) and methods appendix
18	Discuss limitations of the estimates. Include a discussion of any modelling assumptions or data limitations that<br><br>affect interpretation of the estimates.	Discussion of limitations was provided	Main text (Limitations) and methods appendix

© 2025 Dieleman JL et al. JAMA Health Forum.

References

Dwyer-Lindgren, L., Kendrick, P., Kelly, Y. O., Baumann, M. M., Compton, K., Blacker, B. F., Daoud, F., Li, Z., Mouhanna, F., Nassereldine, H., Schmidt, C., Sylte, D. O., Hay, S. I., Mensah, G. A., Nápoles, A. M., Pérez-Stable, E. J., Murray, C. J. L., & Mokdad, A. H. (2023). Cause-specific mortality by county, race, and ethnicity in the USA, 2000–19: a systematic analysis of health disparities. In The Lancet (Vol. 402, Issue 10407, pp. 1065–1082). Elsevier BV. https://doi.org/10.1016/s0140-6736(23)01088-7

Vos, T., Lim, S. S., Abbafati, C., Abbas, K. M., Abbasi, M., Abbasifard, M., Abbasi-Kangevari, M., Abbastabar, H., Abd-Allah, F., Abdelalim, A., Abdollahi, M., Abdollahpour, I., Abolhassani, H., Aboyans, V., Abrams, E. M., Abreu, L. G., Abrigo, M. R. M., Abu-Raddad, L. J., Abushouk, A. I., … Murray, C. J. L. (2020). Global burden of 369 diseases and injuries in 204 countries and territories, 1990–2019: a systematic analysis for the Global Burden of Disease Study 2019. In The Lancet (Vol. 396, Issue 10258, pp. 1204–1222). Elsevier BV. https://doi.org/10.1016/s0140-6736(20)30925-9

Elbers, B. (2024). Shapley. GitHub repository, https://github.com/elbersb/shapley

Shorrocks, A. F. (2012). Decomposition procedures for distributional analysis: a unified framework based on the Shapley value. In The Journal of Economic Inequality (Vol. 11, Issue 1, pp. 99–126). Springer Science and Business Media LLC. https://doi.org/10.1007/s10888-011-9214-z

Das Gupta, P. (1993). Standardization and Decomposition of Rates: A User's Manual, U.S. Bureau of the Census, Current Population Reports, Series P23, n. 186, Washington, D.C.

Das Gupta, P. (1978). A General Method of Decomposiing a Difference Between Two Rates into Several Components. *Demography*, *15*(1), 99–112. https://doi.org/10.2307/2060493

Area Health Resources Files (AHRF) 2020-2021. US Department of Health and Human Services, Health Resources and Services Administration, Bureau of Health Workforce, Rockville, MD.

Ruggles, S., Flood, S., Sobek, M., Brockman, D., Cooper, G., Richards, S., & Schouweiler, M. (2023). IPUMS USA: Version 13.0 (13.0) [Dataset]. Minneapolis, MN: IPUMS. https://doi.org/10.18128/D010.V13.0. Accessed March 8, 2023.

© 2025 Dieleman JL et al. JAMA Health Forum.

US Census Bureau. American Community Survey, 2009–2021 American Community Survey 5-Year Estimates, Table B15001; using Census data portal; https://data.census.gov/cedsci/. Accessed April 26, 2023.

Geocorr Applications - MCDC. https://mcdc.missouri.edu/applications/geocorr.html Accessed Sept 25, 2023.

National Center for Health Statistics, Centers for Disease Control and Prevention, US Census Bureau. United States Vintage 2020 Bridged-Race Postcensal Population Estimates 2010–2020. Hyattsville, United States: National Center for Health Statistics, Centers for Disease Control and Prevention, 2020. https://www.cdc.gov/nchs/nvss/bridged_race.htm. Accessed February 17, 2022.

Bureau of Labor Statistics (United States). United States Consumer Price Index - All Urban Consumers. Washington, D.C., United States of America: Bureau of Labor Statistics (United States).

United States Census Bureau (USCB). United States Small Area Income and Poverty Estimates 2019. Washington, D.C., United States of America: United States Census Bureau (USCB).

Minnesota Population Center, University of Minnesota, United States Census Bureau (USCB). United States American Community Survey 5-Year Estimates 2008-2012:2016-2020 - NHGIS. Minneapolis, United States of America: University of Minnesota, 2021.

Minnesota Population Center, University of Minnesota, United States Census Bureau (USCB). United States Census Housing Units by Urban/Rural Status Time Series 1980-2010. Minneapolis, United States of America: University of Minnesota, 2011.

© 2025 Dieleman JL et al. JAMA Health Forum.
