# Supplementary material for: Drivers of Variation in Health Care Spending Across US Counties
Source: JAMA Health Forum. 2025 Feb 14;6(2):e245220. doi: 10.1001/jamahealthforum.2024.5220 (PMC11829242; doi:10.1001/jamahealthforum.2024.5220)
Supplement: Supplement 2. — Data Sharing Statement [file jamahealthforum-e245220-s002.pdf]

Data Sharing Statement

Dieleman. Drivers of Variation in Health Care Spending Across US Counties. *JAMA Health Forum*. Published February 14, 2025. doi:10.1001/jamahealthforum.2024.5220

Data
Data available: Yes
Data types: Data (not involving human participants), Data dictionary
How to access data: https://ghdx.healthdata.org/
When available: With publication

Supporting Documents
Document types: Statistical/analytic code
How to access documents: https://github.com/ihmeuw/Resource_Tracking_US_DEX
When available: With publication

Additional Information
Who can access the data: anyone requesting the data
Types of analyses: any non-commercial use
Mechanisms of data availability: without investigator support
